# Interventions for Perpetrators of Intimate Partner Violence: An Umbrella Review of Systematic Reviews

**DOI:** 10.1002/ab.70056

**Published:** 2026-01-05

**Authors:** Giulia Punzo, Patrizia Velotti

**Affiliations:** ^1^ Department of Dynamic and Clinical Psychology, and Health Studies, Faculty of Medicine and Psychology Sapienza University of Rome Rome Italy

**Keywords:** interventions, intimate partner violence, IPV, perpetrators, treatments

## Abstract

Intimate partner violence (IPV) represents a global public health concern, with significant psychological, physical, and social consequences. Numerous interventions have been proposed and evaluated over time to address perpetrator behavior; however, the heterogeneity and variability of outcomes across studies limit the clarity of evidence‐based guidance. To address this, the present study conducted an umbrella review of systematic reviews and meta‐analyses, aiming to synthesize and critically appraise the highest level of available evidence on interventions targeting IPV perpetrators. A comprehensive search was conducted across five major databases (PsycInfo, PsycArticles, Scopus, PubMed, and MEDLINE), supplemented by Google Scholar and manual bibliographic screening. The initial search yielded 8827 records. After duplicate removal and screening phases, a total of 41 systematic reviews met the inclusion criteria. The included studies span a wide range of intervention types, target populations, and outcome measures. This umbrella review highlights key methodological limitations in the literature, varying degrees of effectiveness across intervention modalities, and gaps in long‐term outcome evaluations. By providing a high‐level synthesis, the findings offer valuable insights for practitioners, policymakers, and researchers, informing the development of more effective and evidence‐based strategies for the prevention and reduction of IPV perpetration.

## 1 Introduction

Intimate partner violence (IPV) represents a pervasive public health issue, affecting millions of individuals across diverse sociocultural contexts. IPV encompasses a spectrum of harmful behaviors occurring within current or former intimate relationships, including physical aggression, sexual coercion, psychological abuse, controlling conduct, stalking, threats, and economic manipulation (World Health Organization 2012). According to the World Health Organization (2021), approximately 30% of women worldwide have experienced physical and/or sexual violence by an intimate partner in their lifetime, with more than 10 million individuals affected each year. Among its various forms, emotional abuse is particularly widespread and often precedes or co‐occurs with physical violence (Kelly and Payton 2019). Epidemiological evidence indicates higher prevalence rates among adolescents and young adults, individuals from lower socioeconomic backgrounds, and those experiencing unemployment (Barner and Carney 2011; Schumacher and Leonard 2005; Stith et al. 2004). These patterns highlight the intersectionality of risk factors and the need for comprehensive, context‐sensitive interventions to address the multifaceted nature of IPV.

A distinguishing feature of IPV, compared with other forms of interpersonal violence, is that it occurs within emotionally intimate relationships (Velotti et al. 2019; Velotti et al. 2022; Cataudella et al. 2023). The repeated and escalating interactions through which IPV unfolds make its psychological dynamics particularly complex and often render victims unable to fully recognize the danger they face (Capaldi et al. 2012).

Despite extensive research, definitions and theoretical models of IPV vary across disciplines and cultural contexts (Burelomova et al. 2018; Chesworth 2018). Nevertheless, IPV is broadly conceptualized as violence occurring within an intimate relationship (Finkel 2007; Finkel and Eckhardt 2013; Chester and DeWall 2018; Miller and McCaw 2019), with several meta‐theoretical frameworks proposed to elucidate its etiology—among them the I³ Model (Finkel 2014) and the General Aggression Model (DeWall et al. 2011). There is growing consensus recognizing IPV as a major public health concern with profound individual, relational, and societal consequences, necessitating coordinated and urgent intervention (Peterson et al. 2018).

Intervention efforts historically prioritized victim protection—ensuring safety, offering psychological support, and addressing legal and social needs (Barner and Carney 2011). However, the Istanbul Convention (Council of Europe 2011) frames IPV a systemic issue requiring integrated preventive, protective, and prosecutorial strategies. In response, multi‐agency models have been increasingly promoted, emphasizing the coordination of victim services with perpetrator accountability and evidence‐based rehabilitation (Butters et al. 2021; Kelly and Johnson 2008; Stith et al. 2005; Capaldi and Kim 2007). Correspondingly, attention has grown toward the development and evaluation of perpetrator intervention programs aimed at reducing recidivism and promoting long‐term behavioral change (Babcock et al. 2017).

### 1.1 Interventions for Perpetrators

Despite increasing recognition of the need for targeted interventions addressing IPV perpetrators (Manita and Matias 2016), the development and evaluation of effective programs have been hindered by both methodological and conceptual challenges. IPV perpetration encompasses a wide spectrum of abusive behaviors, including physical aggression, psychological and emotional abuse, coercive control, sexual coercion, and threatening or intimidating conduct (Breiding et al. 2015; Garofalo and Velotti 2017; Stark 2007). These behaviors frequently co‐occur and vary considerably across perpetrators, making it difficult to develop interventions that adequately address the heterogeneity of abusive profiles. In parallel, IPV‐related behaviors denote co‐occurring risk factors that do not constitute violence per se but are empirically associated with an increased likelihood or severity of IPV—such as alcohol (Thompson and Kingree 2006; D'Aguanno et al. 2017) and substance abuse (Reardon et al. 2020; Sousa et al. 2024), anger dyscontrol (Garofalo and Velotti 2017) and impulsivity (Rogier et al. 2019). Although these domains are not forms of IPV, they represent clinically meaningful treatment targets that may indirectly reduce the risk or expression of violent behavior.

Across time, various intervention models have been introduced, each grounded in distinct theoretical assumptions (Heyman et al. 2021). Yet these frameworks are often fragmented and insufficiently capture the multifaceted nature of IPV, particularly the substantial heterogeneity among perpetrators and the interplay of individual, relational, and contextual factors shaping abusive conduct (Gondolf and Williams 2001; Murphy and Meis 2008).

One of the most widely implemented frameworks is the Duluth Model, rooted in feminist theory, which conceptualizes IPV as a expression of patriarchal control by men over women (Pence and Paymar 1993). Although some studies report modest reductions in violent behavior following Duluth‐based interventions (Babcock and La Taillade 2000; Miller et al. 2013), meta‐analytic evidence generally indicated limited or non‐significant effects on recidivism (Babcock et al. 2004; Feder and Wilson 2005). Moreover, its primarily psychoeducational structure tends to overlock relevant psychological, motivational, and relational dimensions of IPV (Carney and Buttell 2006; Miller et al. 2013), as well as key clinical factors such as trauma histories, emotional dysregulation, psychiatric comorbidity, and bidirectional violence (Langhinrichsen‐Rohling et al. 2012; Cannon and Buttell 2016; Bates et al. 2017).

Cognitive behavioral therapy (CBT) represents another major framework, focusing on reducing violent behavior through anger management, behavioral regulation, and cognitive restructuring (Nesset et al. 2019). Group‐based delivery is typically adopted for reasons of cost‐effectiveness and to foster interpersonal learning, accountability, and peer cohesion (Babcock et al. 2007).

Acceptance and commitment therapy (ACT), an evolution of cognitive‐behavioral approaches, seeks to increase psychological flexibility by promoting acceptance of internal experiences and commitment to value‐driven behavior (Hayes et al. 1999). Rather than targeting the elimination of negative thoughts or emotions, ACT directly addresses experiential avoidance—a construct strongly linked to aggression (Zarling et al. 2019). Empirical findings suggest that ACT improves psychological flexibility (Zarling et al. 2025) and reduces IPV‐related behaviors among male perpetrators (Thompson and Kingree 2006; Reardon et al. 2020; Berkout et al. 2019).

Motivational interviewing (MI) offers a complementary, client‐centered approach aimed at enhancing motivation for change through empathy, discrepancy development, and the reinforcement of self‐ efficacy (Miller and Rollnick 2012; Austin et al. 2011). MI can be used as a standalone intervention or integrated with other modalities. Evidence indicates that MI enhances treatment engagement and behavioral change, particularly among individuals with low readiness for change (Soleymani et al. 2018).

Findings from systematic reviews and meta‐analyses on IPV remain inconsistent. Many studies report limited or nonsignificant reductions in violent behavior following perpetrator interventions, with effect sizes decreasing as methodological rigor increases (Eckhardt et al. 2006; Butters et al. 2021; Voith et al. 2020). These limitations have contributed to a persistent perception that perpetrator programs are broadly ineffective (Eckhardt et al. 2006). Progress in the field is further impeded by theoretical disputes, limited interdisciplinary collaboration, and inconsistencies in the methodologies used to evaluate program outcomes (Hamberger et al. 2022). The proliferation of overlapping systematic reviews and meta‐analyses only adds to the conceptual confusion, complicating evidence synthesis and hindering translation into practice.

### 1.2 Current Study

Given the substantial heterogeneity observed across primary studies—with respect to intervention types, study designs, populations, and outcome measures—a new systematic review or meta‐analysis of individual studies would likely replicate existing inconsistencies without advancing theoretical or clinical clarity. An umbrella review was therefore deemed the most appropriate approach, as it synthesizes evidence from multiple systematic reviews and meta‐analyses and allows for a higher‐order appraisal of their methodological rigor. Umbrella reviews provide an overarching evaluation of review‐level evidence, enabling comparison of findings across studies and identification of persistent gaps or inconsistencies in the literature (Aromataris et al. 2014). This approach is particularly well suited to IPV perpetration research, given its pronounced heterogeneity and the fragmentation of existing evidence (Papatheodorou 2019).

By systematically compiling and critically evaluating review‐level evidence, the present study aims to clarify which interventions demonstrate the strongest empirical support, identify methodological limitations within the literature, and offer guidance for future research and enhanced clinical practice in the field of IPV perpetrator interventions.

## 2 Method

### 2.1 Search Strategy

A comprehensive umbrella review was conducted within the major databases: PsycInfo, PsycArticles, Scopus, PubMed, and MEDLINE (all years up to February 5, 2025). We adhered to the Preferred Reporting Items for Systematic Review and Meta‐analysis (PRISMA) guidelines (Liberati et al. 2009), ensuring methodological transparency and replicability.

The search strategy employed three key concepts: (1) IPV; (2) Intervention; (3) Review. Search terms were entered using the boolean operators “OR” (within the same construct) and “AND” (to combine the search for the three constructs) and utilizing title or abstract (title/abstract) field codes. The complete list of search terms used within the five databases during the article identification phase are presented in Table 1.

**Table 1:** Search filter.

Construct 1: Intimate partner violence A. Intimate relationship Spous* OR Intimate OR Dating OR Romantic OR Husband* OR Partner* OR Wife OR Wives OR Marital OR Married OR Pregnan* OR Feminicide OR Domestic OR Conjugal* OR Consort* OR Couple* AND B. Violence Abus* OR Aggress* OR Violen* OR Homicide OR Humiliat* OR “controlling behavior” OR “controlling behaviour” OR threat* OR battering OR battered OR offen* OR coertion OR coercitive OR assault* OR maltreat* OR rape* OR beat* OR hurt* OR insult* AND C. Instruments, specific expressions “Domestic Violence” OR “Gender‐based Violence” OR “Intimate Partner Violence” OR IPV OR “gender‐based violence” OR “patriarchal terrorism” OR “Coercive Controlling Violence” OR “Violent Resistance” OR “Mutual Violent Control Violence” OR “Separation‐Instigated Violence” OR “Male‐Controlling Interactive Violence” OR “Conflict Motivated Violence” OR “Episodic male battering” OR “Separation‐engendered violence” OR “Conflict Tactics Scales” OR “Abuse Assessment Screen” OR “Violence Against Women Survey” OR “Sexual Experience Survey” OR “Severity of Violence Against Women” OR “Women's Experience with Battering” OR “Woman Abuse Screening Tool” OR “Composite Abuse Scale” OR “Behavior Risk Factor Surveillance System Module” OR “Norvold Questionnaire” OR “Danger Assessment Scale” OR “Hurt. Insult. Threat. Scream” OR “Psychological Maltreatment of Women Inventory” OR “Humiliation, afraid, rape AND kick” OR “Hurt insulted threatened OR screamed at questionnaire” OR “Humiliation, afraid, rape AND kick” OR “Emotional Abuse Questionnaire” OR “Psychological maltreatment of woman inventory” OR “Psychological maltreatment of partner”
Construct 2: Intervention Intervention OR Treatment OR Therapy OR Counseling OR Education OR Program OR Curriculum OR Assessment OR Recidivism OR Re‐offending OR Evaluation OR Efficacy OR Effectiveness
Construct 3: Review Review OR Overview OR Systematic Review OR Meta‐analysis OR Revision

Grey literature was obtained by conducting a search within Google Scholar (all years up to February 2025) using the following terms: IPV, domestic violence, intervention effectiveness, systematic review, meta‐analysis, and extracting the first ten pages of results, in line with prior recommendations for grey literature searches.

During the initial screening phase, after removing duplicates, all retrieved records were independently screened by two reviewers based on title and abstract, applying predefined inclusion and exclusion criteria. Discrepancies between reviewers were resolved through discussion or consultation with a third reviewer when necessary.

### 2.2 Eligibility Criteria

To be included in this umbrella review, studies must be either systematic reviews or meta‐analysis focusing on the effectiveness of interventions for IPV perpetrators. Nonsystematic literature reviews (e.g., scoping reviews), primary studies, and theoretical articles related to interventions for IPV perpetrators, as well as systematic reviews focusing on prevalence, risk factors, or diagnostic tools in the context of IPV, are excluded from this umbrella review. The interventions examined in the primary studies may take any form or content (e.g., educational, rehabilitative, or therapeutic) as long as they are specifically directed at individuals perpetrating violence within a couple. Judicial interventions (such as restraining orders and removal from the victim) are also included. However, universal preventive interventions, interventions aimed at direct or indirect victims of IPV (e.g., children witnessing violence), and interventions targeting personnel working in the field of IPV perpetrator intervention are excluded.

The umbrella review includes systematic reviews comprised of primary studies with any research design, ranging from observational studies to randomized controlled trials (RCTs), provided they report data on the intervention's effectiveness in reducing or ceasing violence perpetration, or on outcomes related to variables associated with the reduction or cessation of IPV perpetration (e.g., motivation for change, substance abuse). No restrictions are applied regarding the measurement instruments used to assess IPV perpetration, nor regarding perpetrators' gender, sexual orientation, ethnic background, or age.

Mixed reviews which, in addition to primary studies meeting the aforementioned inclusion criteria, identify and analyze studies that do not meet these criteria (such as interventions targeting IPV victims) are included as long as studies focusing on IPV perpetrators are presented separately in the review, allowing for data extraction.

### 2.3 Data Extraction

After completing the initial screening phase, studies that met the inclusion criteria and those where title and abstract reading were insufficient to determine inclusion or exclusion were retrieved and assessed in full text against the same criteria. For each systematic review included at the end of the screening process, the following data were systematically extracted: author, year of publication, journal name, country, number of studies included in the systematic review and/or meta‐analysis, number of subjects, population characteristics (e.g., percentage of male participants, mean age or age range). Other extracted information of the primary studies was extracted such as intervention details (type, duration, follow‐up, dropout rate), source and outcome measure (e.g., self‐report, partner report, official records) and methodological quality of primary studies.

For systematic review that included meta‐analyses, we extracted statistical data regarding the effect size with corresponding confidence intervals, significance level (*p* values), and heterogeneity index (I^2^) were extracted.

### 2.4 Evaluation of Methodological Quality of Systematic Studies

The methodological quality of included systematic studies was assessed using the AMSTAR‐2 tool (A MeaSurement Tool to Assess systematic Reviews, version 2; Shea et al. 2020). This tool is an adapted version of its previous version, AMSTAR (Shea et al. 2007), designed to assess systematic reviews that include both randomized and nonrandomized studies. The quality assessment was conducted by one author trained in systematic review methodologies. To enhance consistency and reduce potential rater bias, 20% of the included reviews were independently appraised by a second researcher. Inter‐rater agreement was high, and any discrepancies were resolved through discussion until consensus was reached.

The AMSTAR‐2 protocol consists of a list of 16 items addressing various aspects of systematic methodology: research question and inclusion criteria, search strategy, study selection and data extraction, risk of bias at the primary study and review level, and appropriateness of meta‐analytic methods. The full list of items is reported at the end of Table 2.

**Table 2:** Quality assessment of systematic studies.

Reviews	1	2	3	4	5	6	7	8	9	10	11	12	13	14	15	16	Overall confidence
Akoensi et al. (2013)	Y	N	Y	Y	N	N	Y	Y	Y/N	N			Y	Y		Y	Low
Arce et al. (2020)	Y	N	Y	Y	N	Y	Y	Y	Y/N	N	Y	Y	Y	Y	Y	Y	Low
Arias et al. (2013)	Y	N	Y	Y	N	Y	Y	Y	Y/N	N	Y	Y	Y	Y	Y	Y	Low
Babcock et al. (2004)	Y	N	Y	N	N	Y	Y	Y/N	Y/N	N	Y	Y	Y	Y	Y	N	Low
Babcock et al. (2024)	Y	Y	Y	Y	Y	Y	N	Y	Y/N	N	Y	Y/N	Y	Y	Y	Y	Moderate
Benitez et al. (2010)	Y	N	N	Y/N	N	N	N	Y/N	Y	N			Y	Y		Y	Low
Cheng et al. (2021)	Y	N	Y	Y	N	N	Y	Y	Y	N	Y	Y	Y	Y	Y	Y	Low
Cordier et al. (2021)	Y	Y	Y	Y	Y	Y	Y	Y	Y	Y	Y	Y	Y	Y	Y	Y	High
Eckhardt et al. (2013)	Y	N	Y	Y	Y	Y	N	Y	N	N			Y	Y		N	Low
Feder et al. (2008)	Y	Y	Y	Y	Y	Y	Y	Y	Y	N	Y	Y	Y	Y	Y	Y	High
Fernández‐Fernández et al. (2022)	Y	N	N	Y	N	Y	Y/N	Y	Y	N	Y	Y	Y	Y	Y	Y	Low
Ferrer‐Perez and Bosch‐Fiol (2018)	N	N	N	Y	N	N	N	Y/N	Y/N	N			Y	Y		Y	Low
Gannon et al. (2019)	Y	Y	Y	Y	N	Y	Y	Y	Y	N	Y	Y	Y	Y	Y	Y	Moderate
Garner et al. (2021)	Y	N	Y	Y	Y	Y	N	N	Y	N	Y	Y	Y	Y	Y	N	Low
Gilchrist et al. (2015)	Y	N	N	Y/N	Y	Y	Y	Y	Y	N			Y	Y		N	Low
Karakurt et al. (2016)	Y	Y	Y	Y	Y	Y	Y	Y	Y	Y	Y	Y	Y	Y	Y	Y	High
Karakurt et al. (2019)	Y	N	Y	Y	Y	Y	Y	Y	Y	N	Y	Y	Y	Y	Y	Y	High
Laskey (2016)	Y	N	Y	N	N	N	N	N	N	N			N	Y		N	Critically low
Lilley‐Walker et al. (2018)	Y	N	Y	Y	Y	Y	Y/N	Y/N	N	N			Y	N		Y	Low
McMurran (2009)	Y	N	Y	Y	N	N	N	N	Y	N			Y	Y		N	Low
Nesset et al. (2019)	Y	Y	Y	Y	Y	Y	Y	Y	Y	N			Y	Y		Y	High
Oğuztüzün et al. (2023)	Y	Y	Y	Y	N	N	N	Y	N	N	Y	N	N	Y	N	Y	Critically low
Pinto e Silva et al. (2023)	Y	Y	Y	Y	Y	Y	N	Y	Y	N			N	Y		Y	Moderate
Roldán‐Pardo et al. (2024)	Y	Y	Y	Y	Y	Y	Y/N	Y	Y	N			Y	Y		Y	High
Santirso et al. (2020)	Y	N	Y	Y	Y	Y	Y	Y	Y	N	Y	Y	Y	Y	Y	Y	Moderate
Satyen et al. (2022)	Y	Y	Y	Y	N	N	N	Y	Y	N	N/A	N/A	Y	Y	N	Y	Low
Smedslund et al. (2007)	Y	Y	Y	Y	Y	Y	Y	Y	Y	N	Y	Y	Y	Y	Y	Y	High
Soleymani et al. (2018)	Y	N	N	Y/N	N	N	N	Y	Y	N			Y	Y		N	Low
Sousa et al. (2024)	Y	Y	Y	Y	Y	Y	N	Y	Y	N	N/A	N/A	Y	Y	N	Y	Moderate
Stephens‐Lewis et al. (2021)	Y	Y	Y	Y	Y	Y	Y	Y	Y	N	Y	Y	Y	Y	Y	N	High
Stith et al. (2021)	Y	N	Y	Y	Y/N	Y/N	N	Y	N	N			N	Y	N	Y	Low
Stjernqvist and Strand (2024)	Y	Y	Y	Y	Y	N	N	Y	Y	N	N/A	N/A	Y	Y	N	Y	Moderate
Stover et al. (2009)	Y	N	Y	Y	N	N	N	Y	N	N			Y	Y		N	Critically low
Tarzia et al. (2020)	Y	Y	Y	Y	Y	N	Y	Y	Y	N			Y	Y		Y	Moderate
Travaini et al. (2024)	Y	Y	Y	Y	Y	Y	Y	Y	Y	N			Y	Y		Y	High
Travers et al. (2021)	Y	Y	Y	Y	N	N	Y	Y	Y	N	Y	Y	Y	Y	Y	Y	Moderate
Vall et al. (2024)	Y	Y	Y	Y	N	N	N	Y	Y	N			N	Y		Y	Moderate
Waller (2016)	Y	N	N	Y	N	N	N	Y	Y	N			Y	Y		N	Critically low
Wilson et al. (2021)	Y	Y	Y	Y	Y	Y	Y	Y	Y	N	Y	Y	Y	Y	Y	Y	High
	Y	N	Y	Y	N	Y	Y	Y/N	N	N			Y	Y		Y	Low
Wynter et al. (2025)	Y	Y	Y	Y	Y	N	N	Y	N	N			N	Y		Y	Moderate

*Note:* 1. Did the research questions and inclusion criteria for the review include the components of PICO? 2. Did the report of the review contain an explicit statement that the review methods were established prior to the conduct of the review and did the report justify any significant deviations from the protocol? 3. Did the review authors explain their selection of the study designs for inclusion in the review? 4. Did the review authors use a comprehensive literature search strategy? 5. Did the review authors perform study selection in duplicate? 6. Did the review authors perform data extraction in duplicate? 7. Did the review authors provide a list of excluded studies and justify the exclusions? 8. Did the review authors describe the included studies in adequate detail? 9. Did the review authors use a satisfactory technique for assessing the risk of bias (RoB) in individual studies that were included in the review? 10. Did the review authors report on the sources of funding for the studies included in the review? 11. If meta‐analysis was performed, did the review authors use appropriate methods for statistical combination of results? 12. If meta‐analysis was performed, did the review authors assess the potential impact of RoB in individual studies on the results of the meta‐analysis or other evidence synthesis? 13. Did the review authors account for RoB in primary studies when interpreting/discussing the results of the review? 14. Did the review authors provide a satisfactory explanation for, and discussion of, any heterogeneity observed in the results of the review? 15. If they performed quantitative synthesis did the review authors carry out an adequate investigation of publication bias (small study bias) and discuss its likely impact on the results of the review? 16. Did the review authors report any potential sources of conflict of interest, including any funding they received for conducting the review? (AMSTAR ‐2 tool; Shea et al. 2020).

Each item requires assigning one of the following possible responses: “Yes” (Y), “No” (N), “Partial Yes” (Y/N). The evaluation of each item then contributes to an overall score indicating the quality assessment of the review (overall confidence). The 16 items are distinguished between non‐critical items and critical items (2, 4, 7, 9, 11, 13, 15), with critical items carrying more weight in determining the final assessment. Overall confidence is rated as “high” when there is no or only one noncritical weakness, “moderate” when there is more than one noncritical weakness, “low” if a critical flaw is identified, with or without noncritical weaknesses, and finally “very low” when more than one critical flaw is present with or without noncritical flaws.

## 3 Results

The search across databases (PsycInfo, PsycArticles, Scopus, PubMed, MEDLINE) identified 8618 entries, with an additional 209 records extracted from the Google Scholar search, totalizing 8827 results. After removing 6164 duplicates, the first phase of record screening examined 2464 entries based on title and abstract. Of these, 2307 were excluded for not meeting the inclusion criteria, while 157 studies were retained for full‐text screening. Within this group, the manuscripts of 5 documents could not be retrieved, while the remaining 152 were evaluated in their entirety against the inclusion criteria, resulting in the identification of 32 eligible systematic studies. Through additional bibliographic screening of included studies and targeted web searches, 15 more entries were identified, of which 9 met the eligibility criteria.

At the end of the screening process, a total of 41 systematic studies were included in this umbrella review. Notably, one study appears in two publications, an official report (Hamilton et al. 2013), and a scientific publication (Akoensi et al. 2013). Another study is featured in three different publications, two scientific articles (Feder and Wilson 2005; Feder and Wilson 2005), and a book chapter (Feder and Wilson 2005).

**Figure 1:**
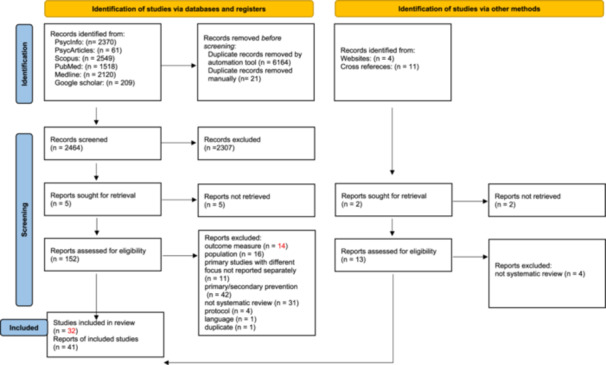
Flow diagram (Page et al. 2021). [Color figure can be viewed at wileyonlinelibrary.com]

The study selection process is fully illustrated in the PRISMA flow diagram (Figure 1).

Twenty‐thre out of the 41 included studies were systematic reviews, while the remaining were meta‐analyses (18). Among the meta‐analytic studies, two are updates of meta‐analyses already included in previous reviews (Arce et al. 2020; Wilson et al. 2021).

### 3.1 Systematic Reviews

#### 3.1.1 Characteristics of the Studies

**Figure 2:**
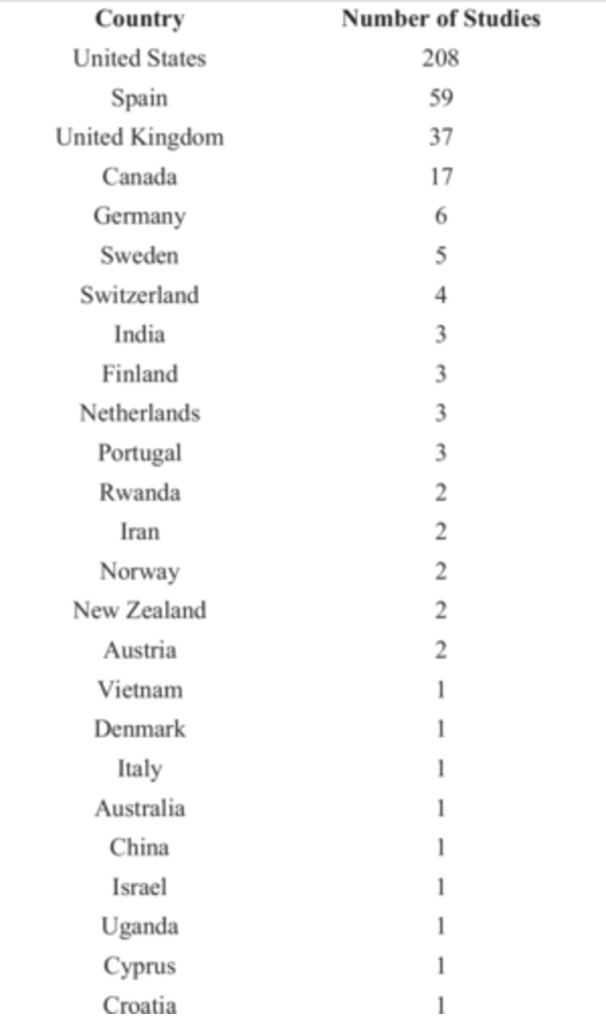
Geographic origin of primary studies (systematic reviews).

The 23 systematic reviews were published in scientific journals between 2009 and 2025. They include 365 primary studies conducted between 1984 and 2022 in diverse geographical regions, predominantly conducted in the United States (57%), Spain (16%), and the United Kingdom (10%) (See Figure 2). The reviews were conducted in the United States (5), the United Kingdom (3), Australia (3), Spain (2), Portugal (2), New Zealand (1), Italy (1) or in collaboration with multiple countries (6), including Canada, Norway, Germany, and Sweden among others.

Most systematic reviews assessed the effectiveness of interventions in reducing physical IPV, which represented the most consistently reported outcome across primary studies. In addition to physical aggression, several reviews also examined psychological/emotional abuse (Ferrer‐Perez and Bosch‐Fiol 2018; Nesset et al. 2019; Stith et al. 2004; Waller 2016) or verbal aggression (Akoensi et al. 2013), while a smaller number evaluated combined outcomes that included physical, psychological, and verbal forms of IPV (Sousa et al. 2024; Tarzia et al. 2020). A few studies also examined the effectiveness of interventions directed at variables correlated with IPV perpetration, such as stalking behaviors (Travaini et al. 2024), alcohol abuse (Pinto e Silva et al. 2023), motivation to change (McMurran 2009; Soleymani et al. 2018), treatment adherence (Roldán‐Pardo et al. 2024) and parenting behaviors (Wynter et al. 2025). These studies explored the moderating effect of such variables on IPV perpetration. Within each systematic review, there are between 4 and 60 primary studies, and the total sample size of the systematic reviews is of 153,555 participants.

#### 3.1.2 Research Designs

Regarding the research design, four systematic reviews (Stover et al. 2009; Gilchrist et al. 2015; Pinto e Silva et al. 2023) presented only randomized clinical trials (RCTs). Most systematic studies included experimental (RCTs) and quasi‐experimental studies (Eckhardt et al. 2013; Stith et al. 2004; Satyen et al. 2022; Vall et al. 2024; Travaini et al. 2024; Roldán‐Pardo et al. 2024; Sousa et al. 2024; Wynter et al. 2025). Four systematic reviews included only quasi‐experimental studies (Akoensi et al. 2013; Ferrer‐Perez and Bosch‐Fiol 2018; McMurran 2009; Stjernqvist and Strand 2024), and the remaining systematic studies included descriptive and observational non‐experimental studies (Benitez et al. 2010; Laskey 2016; Waller 2016; Lilley‐Walker et al. 2018; Soleymani et al. 2018; Tarzia et al. 2020).

Only one review (Nesset et al. 2019) included both RCTs, quasi‐experimental, descriptive and observational non‐experimental studies.

#### 3.1.3 Quality Assessment of Systematic Reviews

Table 2 presents the quality assessment of systematic studies, concerning each question and the overall reliability assessment. Regarding systematic reviews, 11 reviews received a rating of “low” overall reliability (McMurran 2009; Benitez et al. 2010; Akoensi et al. 2013; Eckhardt et al. 2013; Gilchrist et al. 2015; Ferrer‐Perez and Bosch‐Fiol 2018; Soleymani et al. 2018; Lilley‐Walker et al. 2018; Stith et al. 2004; Satyen et al. 2022), while six were rated “moderate” (Tarzia et al. 2020; Pinto e Silva et al. 2023; Sousa et al. 2024; Stjernqvist and Strand 2024; Vall et al. 2024; Wynter et al. 2025), three were rated “high” (Nesset et al. 2019; Roldán‐Pardo et al. 2024; Travaini et al. 2024), and three were rated “critically low” (Stover et al. 2009; Laskey 2016; Waller 2016).

Overall, the most critical weaknesses concerned the explicit formulation of a systematic methodology and research question within a predefined protocol, as well as the detailed and comprehensive description of included and excluded studies and an explicit assessment of bias risks.

#### 3.1.4 Sample Characteristics

Across the systematic reviews, the study populations display substantial heterogeneity, with the notable exception of participant gender. The majority of the samples are composed exclusively of male perpetrators, with the exception of one review focusing solely on female participants (Laskey 2016) and four reviews (Lilley‐Walker et al. 2018; Wynter et al. 2025; Travaini et al. 2024; Sousa et al. 2024) that include studies involving both male and female perpetrators. All reviews target adult populations; however, only a minority report the average age of participants (see Table 3).

**Table 3:** Features systematic reviews.

				Population					
References	Name of journal	Country	N. of primary studies	N. of subject	Gender (% maschi)	Age	Research design	Type of intervention	Overall confidence (AMSTAR)
Akoensi et al. (2013)	*International Journal of Offender Therapy and Comparative Criminology*	United Kingdom Germany	12	1833	100	nr	Quasi‐experimental	Duluth CBT Duluth + CBT Psychoeducational Psychodynamic	Low
Benitez et al. (2010)	*The Journal of the American Academy of Psychiatry and the Law*	United States	15	40,833	nr	nr	Control retrospective retrospective prospectic descriptive observational	Protection orders	Low
Eckhardt et al. (2013)	*Partner Abuse*	United States	30	23,287	100	33,4	RCT quasi‐experimental	Duluth CBT CBT culturally focused (African American) Anger management MI Couples therapy IPV + SU	Low
Ferrer‐Perez and Bosch‐Fiol (2018 *)*	*International Journal of Offender Therapy and Comparative Criminology*	Spain	13	4181	100	nr	Pre‐post control pre‐post ex‐post‐facto	Duluth CBT CBT adjusted Ecological model Emotional treatment mindfulness ACT	Low
Gilchrist et al. (2015)	*Aggression and Violent Behavior*	United Kingdom Spain United States	4	2426	100	nr	RCT	CBT + anger management	Low
Laskey (2016)	*Journal of applied Psychology and Social Science*	United Kingdom	8	1364	0	nr	Pre‐mid‐post pre‐post descriptive qualitative	Psychoeducational MI + feedback sessions for couples IPV intervention	Critically low
Lilley‐Walker et al. (2018)	*International Journal of Offender Therapy and Comparative Criminology*	United Kingdom	60	8062	96.4	nr	RCT quasi‐experimental nonexperimental	Duluth CBT CBT/psychoeducational/femminist CBT + SU Emotional treatment IPV + SU Ecological model Psychodynamic Restorative Justice Integrative	Low
McMurran (2009)	*Legal and Criminological Psychology*	United Kingdom	13	157	100	nr	Comparison pre‐post	MI MI for SU	Low
Nesset et al. (2019)	*BMC Psychiatry*	Norway Sweden	6	1585	100	34–40	RCT quasi‐experimental control cohort retrospective	CBT CBT + MI CBT/psychoeducational/femminist	High
Pinto e Silva et al. (2023)	*Trauma, Violence & Abuse*	Portugal	15	1785	nr	37	RCT	MI IMP CBT SOCMI BAI SBI SBIP	Moderate
Roldán‐Pardo et al. (2024)	*Trauma, Violence & Abuse*	Spain	13	1254	100%	nr	RCT Observational studies Nonrandomized interventions	CBT Duluth Model MI Psychoeducational Programs	High
Satyen et al. (2022)	*International Journal of Environmental Research and Public Health*	Australia	10	1947	100%	38.8 (4 studies nr)	RCT Pre‐post studies Qualitative design Qualitative observation study Clinical case	CBT Psycho‐educational Programs Duluth Model Transformative gender‐norms interventions New Roots Intervention	Low
Soleymani et al. (2018)	*Aggression and Violent Behavior*	New Zeland	5	841	nr	35.1	RCT quasi‐experimental case study	MI before IPV treatment	Low
Sousa et al. (2024)	*Trauma, Violence & Abuse*	Portugal	23	4086	89%	36,84	RCT Quantitative descriptive Quantitative nonrandomized Mixed method	CBT Motivational, cognitive, and dialectical behavioral therapies MI BT Attachment, family systems therapy Alcohol intervention	Moderate
Stjernqvist and Strand (2024)	*Criminal Justice and Behavior*	Australia	11	7149	nr	36	Quasi‐experimental Retrospective Longitudinal	Arrest IPV court	Moderate
Stith et al. (2021)	*Journal of Marital and Family Therapy*	United States	9 (IPV section only)	2182	50% (1 nr)	nr	RCT Quasi‐experimental	BCT CHRP CTS IBT SAH‐C	Low
Stover et al. (2009)	*Professional Psychology: Research and Practice*	United States	12	9938	100	nr	RCT RCT 2 active treatment	Arrest Duluth Duluth + probation CBT Psychoeducational Couples therapy Group couples therapy	Critically low
Tarzia et al. (2020)	*Trauma, Violence & Abuse*	Australia United Kingdom	13	2245	100	nr	RCT observational	CBT CBT + SU CBT group for veterans (trauma‐informed) Couples therapy Couples therapy + SU MI Alcohol abuse intervention Pharmacological treatment	Moderate
Travaini et al. (2024)	*International Review of Psychiatry*	Italy	10	661	97.9%	29,91	RCT Case series Cohort study Case‐report	CBT DBT ACT Psychodynamic therapy Pharmacological interventions (antipsychotics, SSRIs) Integrated treatment models (combining therapy and medication)	High
Vall et al. (2024)	*Trauma, Violence & Abuse*	Spain Germany United Kingdom	46	15,705	100%	37	RCT Quasi‐experimental Pre‐post studies Control group studies	CBT Duluth Model ACT Psychoeducational programs Attachment‐based interventions Risk–Needs–Responsivity Model Experiential‐Based Psychotherapy	Moderate
Waller (2016)	*Aggression and Violent Behavior*	United States	26	10,809	100	nr	Experimental longitudinal	Duluth CBT Psychoeducational Goal setting	Critically low
	*BMC Public Health*	Australia Canada	4	559	100	30	RCT	Couples MI IPV + alcohol CBT + SU Motivational enhancement therapy	Low
Wynter et al. (2025)	*Trauma, Violence & Abuse*	Australia	15	10,666	66% (4 nr)	29,45	RCT CRT CT Pre‐post studies	REAL GATHER Bandebereho Intervention CCP Sugira Muryango Intervention Family Foundations Program Caring Dads Within Our Reach F4C CBT	Moderate

Abbreviations: ACT, acceptance and commitment therapy; ASMT, anger self‐management training; BAI, brief alcohol intervention; BCT, behavioral couples therapy; BIP, batterer intervention program; BT, behavioral therapy; CAT, cognitive analytic therapy; CBT, cognitive‐behavioral therapy; CCP, couple care for parents of newborns; CTS, conflict tactics scale; DBT, dialectical behavior therapy; F4C, fathers for change; FORNET, forensic offender rehabilitation narrative exposure therapy; GATHER, greeting, ask, tell, help, explain, and refer; IBM‐H, interpretation bias modification‐hostility training; IBT, individually based treatment; IMP, individualized motivational plan; IPV, intimate partner violence; ME, motivational enhancement therapy; MI, motivational interviewing; REAL, responsible, engaged, and loving fathers initiative; RNR, risk‐need‐responsivity model; SADV, standardized aggression and domestic violence treatment; SAH‐C, strength at home‐couples program; SAH‐M, strength at home‐men's program; SBI, standard batterer intervention; SBP, standard batterer program; SOCMI, stages of change motivational interviewing; SU, substance use; SU treatment.

The main descriptive characteristics of the perpetrator populations across the included systematic reviews encompass both voluntary and court‐mandated participation, military background, and the presence of alcohol or substance abuse comorbidities. While three reviews explicitly report psychiatric disorders among sample characteristics (Travaini et al. 2024; Vall et al. 2024), one review focused exclusively on studies with predominantly African American perpetrator samples (Waller 2016), and another included only studies centered on father‐perpetrators (Wynter et al. 2025). Notably, only one review included participants (from a single primary study) identified as perpetrators with a psychological condition related to posttraumatic stress disorder (Tarzia et al. 2020), and one systematic review encompassed studies involving IPV perpetrators who also engaged in stalking behaviors (Travaini et al. 2024) (see Table 4).

**Table 4:** Systematic review.

		Treatment					Outcome		
References	Characteristics of the population*	Model of intervention*	Duration	Setting*	Follow‐up	Attrition rate (%)	Measure*	Source**	Main results
Akoensi et al. (2013)	IPV perpetrators − Court‐mandated − Voluntary − Remand − Not specified	Duluth CBT Duluth + CBT Psychoeducational Psychodynamic	20 weeks 3–30 sessions	− Group − Individual − Group + individual	End of treatment −12 months	8–73	− Criminal justice measures − Rates of physical/verbal abuse − Victim's perception	− Police report − Partner report − Self report − Psychological change assessment	− Nonsignificant positive effect for all treatment − Low methodological quality selection bias: High drop‐out rates→systematic review could not reveal definitive conclusions regarding the effective delivery of domestic violence perpetrator programs in Europe
Benitez et al. (2010)	IPV perpetrators	Protection orders	nr	nr	nr	nr	− Protection order violation rate − Nature of violation	Official record	− Rates of protection order violation between 7.1 and 81.3% − violation associated with: Amount of time since placement of the order, characteristics of the victim and abuser, nature of relationship and nature of abuse, stalking, legal system factors − Low methodological quality: Selection bias, outcome measure, short follow‐up periods →evidence is insufficient to draw conclusions about the effectiveness of probation orders to reduce the risk of violence
Eckhardt et al. (2013)	*IPV perpetrators* − Court‐mandated − Voluntary	Duluth CBT CBT culturally focused (African American) Anger management MI Couples therapy IPV + SU	8–52 sessions	− Group − Couple − Individual − Couple + individual	End of treatment ‐54 months	nr	− IPV recidivism − Re‐arrest for IPV − Re‐offense for any violent behavior − IPV charges	− Official record − Self‐report − Partner report − Self + partner report	− BIP traditional: − No evidence of effectiveness relative to a no‐treatment control group if excluded studies with notable methodological flaws − BIP alternative: Positive effects of “motivational strategies” on change‐relevant attitudes, treatment engagement, and/or abusive behavior. − Low methodological quality: Sample size, high attrition rates of partner follow‐up → evidence is insufficient to draw conclusions about the effectiveness of traditional and alternative BIP
Ferrer‐Perez and Bosch‐Fiol (2018)	IPV perpetrators − Court‐mandated − Voluntary − Voluntary in prison	Duluth CBT CBT adjusted Ecological model Emotional treatment mindfulness ACT	15 sessions ‐1 year	− Group − Individual − Group + individual	End of treatment −18 months	5.7–57	− Improvement in psychological and psychopathological variables − Improvements in attitude toward IPV – treatment completion and motivation to change − Physical/psychological IPV recidivism − Recidivism	− Self‐report − Official record − Partner report − Staff report	− Outcome measures: Psychological/psychopathological variables in Spanish studies − Methodological limitations of outcome studies preclude to determine the effectiveness of interventions: High drop‐out rates, heterogeneity of outcome measures →no significative effects of interventions for perpetrators in Spain
Gilchrist et al. (2015)	Physical IPV perpetratorsmale − Court‐mandated − US Navy military − Alcohol abuse	CBT + anger management	12–26 sessions + 6 monthly meetings	Group	6–12 months	17–71.8	− Physical IPV recidivism − Alcohol use − Alcohol use at baseline	Self/partner report CTS‐2	− Methodological limitations of outcome studies preclude to determine the effectiveness of interventions: Small sample size, low treatment completion, short follow‐up, small number partner report, heterogeneity of duration of intervention, alcohol, and anger espression measure (absent) → evidence is insufficient to draw conclusions about the effectiveness of CBT + anger management interventions
Laskey (2016)	IPV perpetratorsfemale − Court‐mandated − Court‐mandated and voluntary − Coluntary couples	Psychoeducational MI + feedback sessions for couples IPV intervention	nr	nr	nr	nr	− Psychopathological and psychological variables − Relationship variables − Recidivism	− Official record − CTS‐R − IDI − URICA‐DV ‐PCQ − EAQ − TSC‐40 − PAS − PASPH − GCS − ICS − RSE − IMS ‐IFS ‐ASES − ANSIE − SRIS	− Treatment programs provided for female offenders of IPV were originally developed for male perpetrators − Being mandated or nonmandated to treatment had no effect on treatment outcomes − Women seemed to benefit more than men from therapist empathy and open‐ended questions − Only one study IPV recidivism after treatment → evidence is insufficient to draw conclusions about the effectiveness of the intervention with female perpetrators
Lilley‐Walker et al. (2018)	IPV perpetrators − Male − Male and female − Court‐mandated − Voluntary − Court‐mandated and voluntary	Duluth CBT CBT/psychoeducational/femminist CBT + SU Emotional treatment IPV + SU Ecological model Psychodynamic Restorative Justice Integrative	nr	− Group − Group + individual − Community‐base − Prison‐based	1–42 months	nr	− Recidivism − Use of violence and attitude towards women − Perception of risk − Psychopathological − and psychological variables − Relationship variables − Satisfaction with treatment − Motivation to change − SU	− Self‐report − Partner/family report − Police record − Staff report	− European studies show important methodological limitations: Lack of control group, reporting of information about the sample, attrition, and points of time used to collect data
McMurran (2009)	IPV perpetrators − Court‐mandated	MI MI for SU	1–2 sessions	Community‐based	nr	52 (solo uno studio)	− Recidivism − Motivation to change − State of change − Treatment completion	− Self report − Staff report	− Mixed or no significant effects of MI intervention for: Treatment adherence, motivation to change and change in abusive behavior in IPV perpetrators
Nesset et al. (2019)	IPV perpetratorsmale − Court‐mandated − Voluntary − Veterans	CBT CBT + MI CBT/psychoeducational/ feminist	12–26 weeks 2 h per sessions	− Group − Health services − Community setting − Prison/probation − University setting	End of treatment ‐4.6 years	Nr	− IPV recidivism ‐Physical/psychological IPV secondary outcome: ‐Mental health ‐motivation to change − Relationship adjustment	− Self‐report − Partner report − Official record − CTS/CTS2 − URICA − IRI − DAS (1) − MMEA − DAS (2) − Spouse verbal problem checklist	− Methodological limitations of primary studies: Small sample, single source of measurement (self‐report) − Poor use of psychological/psychopathological outcome measures → insufficient scientific evidence to determine effectiveness of group CBT for perpetrators of IPV
Pinto e Silva et al. (2023)	IPV perpetrators	Motivational Interviewing (MI) Individualized Motivational Plan (IMP) Standard Batterer Intervention Program (SBIP) Individual Cognitive Behavioral Therapy (ICBT) Group Cognitive Behavioral Therapy (GCBT) Stages‐of‐Change Motivational Interviewing (SOCMI) Brief Alcohol Intervention (BAI) Motivational Enhancement Therapy (MET) Alcohol Education (AE) Standard Batterer Intervention (SBI)	12–35 weeks	Community setting Prison setting	6–15 months	44% (reported in only 2 studies)	− Recidivism reduction − Treatment compliance − Motivation for change − Aggression and IPV reduction − Interpersonal skills − Anger expression, empathy, psychological outcomes	− Self‐reports − Partner reports − Therapists's assessment − Police and official records	− MIT/IMP significantly increased treatment engagement, motivation and readiness to change − MIT enhanced empathy and decreased aggression − Brief Alcohol Interventions (BAI) helped reduce alcohol use but with limited impact on IPV long‐term − Recidivism rates generally low, with some improvements reported in SBIP + IMP groups
Roldán‐Pardo et al. (2024)	IPV perpetrators	CBT Duluth Model Motivational Interviewing Psychoeducational Programs	16–32 weeks	− Community‐ based interventions − Court mandated programs − Group therapy setting	6 months to 3 years	35%	− Recidivism reduction − Treatment adherence and engagement levels − Facilitator and group dynamics impact on IPV reduction − Psychological variables	− Self‐reports by perpetrators − Facilitator‐reported data − Court and official records − Victim reports	− Group cohesion and structured facilitation were linked to lower physical abuse rate upon follow‐up − Motivational Interviewing combined with CBT led to higher treatment engagement and lower dropout rates − Group leader behavior impacted participant engagement, with directive or rigid facilitators linked to poorer outcomes
Satyen et al. (2022)	IPV perpetrators	Cognitive Behavioral Therapy (CBT) Psycho‐educational Programs Duluth Model Transformative gender‐norms interventions New Roots Intervention	12–52 weeks	− Prison setting − Community setting	nr	nr	− Recidivism − Behavior change − Gender‐related attitude − Family communication − Improvement in psychopathology symptoms	− Self reports − Partner reports − Government‐managed crime registries	− 9/10 studies showed positive outcomes associated with engagement in culturally specific interventions − 6/10 studies reported either a complete absence or reduction in episodes of abuse − Programs implemented in languages and cultural engagement relevant to the client group can enhance client participation and reduce their attrition
Soleymani et al. (2018)	IPV perpetrators	MI before IPV treatment	1 sessions ‐6 weeks	nr	6 months	nr	− Treatment completion − Working alliance − Homework compliance − Number of sessions attended ‐Help seeking behavior − Responsability toward abusive behavior − IPV recidivism	− Self‐report − Partner report − Official record − staff report − Assignment Compliance Rating Scale − WAI − CTS	− Almost all studies show effectiveness of the intervention for adherence to treatment and no significant effectiveness in reducing recidivism (6 months follow‐up) − Methodological limitations primary studies:Heterogeneity in MI conceptualization and outcome measures, lack of longitudinal data →no significant efficacy of the MI intervention before IPV treatment in reducing recidivism →promising results of the MI intervention for treatment adherence
Sousa et al. (2024)	‐IPV perpetrators with SU or alcohol abuse ‐Heterosexual couples	CBT = Cognitive Behavioral Therapy Motivational, cognitive and dialectical behavioral therapies MI = Motivational Interviewing BT = Behavioral Therapy Attachment, family systems therapy Alcohol intervention	12–56 weeks	− Outpatient facilities − Community setting − Inpatient facilities	Nr	55.04%(reported in only 5 studies)	− Substance use − Physical, verbal, or psychological abuse episodes − Recidivism − Attitude toward violence − Risk of recidivism − Social support − Psychopathological symptoms	− Self reports − Police reports − Partner reports − Arrests records − Toxicology screens and breathalyzer results	− SADV showed reduction in alcohol abuse and violence reduction during treatment − BCT, BMT, SBP + BAI reduce physical aggression and better results for those who stop using substances − ADVANCE, I‐StoP, Fathers for Change, ICBT showed improvements in self‐management, physical and verbal IPV, mental health, and alcohol use—some without significant group differences − Context reduced alcohol use, recidivism risk, and depression. MET improved motivation but showed no significant effects on violence or alcohol. CI had no major effects except benefits for those with alcohol abuse.
Stjernqvist and Strand (2024)	IPV perpetrators (mostly male)	Police arrest IPV specialized courts GPS monitoring	nr	Criminal justice Community settings	6 months to 8 years	nr	Official recidivism records, police reports, victim surveys	− Law enforcement records − Rearrest rates − Reports from victims − Re‐abuse rates	Mixed results: Arrest sometimes reduced recidivism short‐term but not long‐term; specialized courts showed some positive effects; limited data on GPS; prosecutorial interventions missing.
Stith et al. (2021)	Couples with low to moderate IPV	BCT Creating Healthy Relationships Programs (CHRP) Conflict Tactics Scale (CTS) Individually Based Treatment (IBT) Strenght at Home Couples Program (SAH‐C)	nr		6 months to 18 months	nr	− IPV recidivism − Reduction of behaviors of propensity toward violence − Prevention of first occurrence of physical abuse	− Conflict Tactics Scale − SPAFF (Specyfic Affect Coding System) − Interviews − Self reports	− Relational education programs are considered probably efficacious interventions. − ePREP (Braithwaite et al. 2011; Braithwaite and Fincham 2014): Significantly reduced physical and psychological IPV vs. active control. − CHRP: Contradictory results from significant IPV reduction to no significant reduction. − Couple CARE for Parents: No IPV reduction; IPV increased among high‐risk participants. − Young Parenthood: Short‐term IPV reduction; effect disappeared at 18‐month follow‐up. − PREP: Long‐term IPV reduction in earlier trials.
Stover et al. (2009)	IPV perpetrators couples − Military − Male partner alcohol abuse	Arrest Duluth Duluth + probation CBT Psychoeducational Couples therapy Group couples therapy	nr	− Group − Couple individual − Couple group	6–18 months	29.3–32.2 15–89 (follow‐up partner)	Recidivism	− Police + partner report − Self + partner report − Partner report − Partner + self‐report + official record − CTS	− Group intervention shows no or minimal effectiveness with respect to arrest − Couple intervention (dyadic and group) shows promising preliminary results for perpetrators with alcohol/substance abuse − Recidivism rate 20%–30% in the 6 months postintervention regardless of the intervention strategy − Lack of studies with long follow‐up →absence of significant effectiveness of group intervention for IPVperpetrators →positive preliminary results for couple intervention for IPV perpetrators with alcohol/substance use
Tarzia et al. (2020)	IPV perpetrators Male − Alcohol abuse − Military − Military with PTSD couples	CBT CBT + SU CBT group for veterans (trauma‐informed) Couples therapy Couples therapy + SU MI Alcohol abuse intervention Pharmacological treatment	1−22 weeks 1−32 sessions	− Alcohol/SA treatment facility‐veterans affairs treatment center ‐hospital psychiatric outpatient facility ‐academic center	End of treatment ‐12 months	0–96 (in media 36.5)	− IPV recidivism ‐Alcohol abuse ‐Help seeking behavior	− CTS − MMEA − TLFB − OAS − Index of Wife Abuse − India Demographic Health Survey (items of physical and sexual violence)	− Paucity of intervention studies for male perpetrators of IPV (absence of intervention studies for male victims of IPV) in healthcare setting − Methodological limitations primary studies: Problematic measures of IPV with CTS and absence of measurement of psychological IPV and verbal aggression in studies, absence of long follow‐up →insufficient scientific evidence to determine effectiveness of intervention for perpetrators of IPV in healthcare settings →promising preliminary results for psychological therapies + alcohol treatment
Travaini et al. (2024)	− Stalking offenders − IPV Perpetrators − Psychiatric disorders − Criminal background	− Cognitive Behavioral Therapy (CBT) − Dialectical Behavior Therapy (DBT) − Acceptance and Commitment Therapy (ACT) − Psychodynamic therapy − Pharmacological interventions (antipsychotics, SSRIs) − Integrated treatment models (combining therapy and medication)	8 weeks to 12 months	− Outpatient forensic psychiatric services − Correctional facilities (prisons) − Community‐based intervention programs − Inpatient psychiatric hospitals	6–36 months posttreatment	15%–55%	− Reduction in stalking behaviors − Changes in attitudes toward violence and control over victims − Reduction in recidivism (re‐arrest, new IPV‐related offenses) − Psychopathological improvement (anxiety, depression, impulsivity)	− Self‐reports by offenders − Police and court records − Victim reports − Psychiatric and forensic evalutation	− CBT and DBT‐based programs showed the strongest reduction in stalking behaviors and recidivism − Pharmacological treatments reduced obsessive behaviors but only combined with psychotherapy − Psychodynamic therapy alone had limited effectiveness in reducing recidivism − Integrated interventions (therapy + medication) had the lowest recidivism rates − Programs targeting emotion regulation (DBT, ACT) led to improvements in anger management and impulse control
Vall et al. (2024)	IPV perpetrators − Substance Abusers − People with psychiatric disorders	Cognitive Behavioral Therapy (CBT) Duluth Model Acceptance and Commitment Therapy (ACT) Psychoeducational programs Attachment‐based interventions Risk–Needs–Responsivity Model Experiential‐Based Psychotherapy Mixed models (combining multiple approaches)—32.6% of studies	8–52 weeks 10–30 sessions	− IPV Perpetrators Programs − Healthcare facilities − Correctional institutions	3–36 months postintervention	40% on average	− IPV recidivism rates − Change in aggressive behaviors − Improvements in anger control and emotional regulation − Reduction in substance use − Enhanced communication skills in relationships	− Self‐reports from perpetrators − Reports from victims/partners − Official data (police records, court reports)	− CBT and Motivational Interviewing showed the best results in reducing recidivism − Duluth Model had mixed results, showing no significant improvements − Short‐term programs (< 12 weeks) showed no significant impact on recidivism − Longer follow‐up periods were associated with greater reductions in recidivism − Regular attendance and program completion significantly reduced IPV perpetration − The use of multiple sources to assess recidivism showed more reliable results
Waller (2016)	Perpetratori IPV male—African American (46%)—Other ethnicities	Duluth CBT Psychoeducational Goal setting	nr	Group community‐based	Up to 48 months	60.6 (globale) 51 (campione afroamericano)	− Physical and nonphysical IPV recidivism − Not specified	Partner report + new arrest record	− Higher dropout rates in the African American population − Only postmodern (alternative) study shows lower rates of dropout and recidivism − Methodological limitations of primary studies: Absence of a standardized definition of attrition (treatment abandonment) → promising preliminary results for alternative intervention models in reducing dropout rate
	IPV prepetrators male − Court‐mandated alcohol abusers − Voluntary with SU − Alcohol abusers arrested for DV − Arrested per DV Couples of university students with male violence	Couples MI IPV + alcohol CBT + SU Motivational enhancement therapy	1 session ‐40 h	− University − BIP − Community setting − SA outpatient treatment	9–12 months	21 (solo uno studio)	− Physical and psychological aggression recidivism − New arrest ‐alcohol use − SU − Mediation of alcohol use in IPV	− Self‐report − Partner report − Record ufficiale − CTS‐2 ‐AUDIT − TFFB − PDAAD − PHDD − Daily drinking questionnaire ‐Breath analyzer − Urine toxicology	− Significant effect for integrated individual and couple intervention for IPV and alcohol on both outcome measures, but results not sustained over time for individual format − No evidence of mediation of alcohol use in the perpetration of IPV → absence of significant effectiveness of treatment for alcohol abuse in perpetrators of IPV
Wynter et al. (2025)	− IPV perpetrators − Fathers at risk to perpetrate IPV	− Psychoeducational programs − Cognitive Behavioral Therapy (CBT)‐based programs − Motivational Interviewing − Transformative gender‐norm interventions − Attachment‐based interventions − Integrated parenting and IPV programs	4 weeks to 12 months	− Community‐based settings − Healthcare settings − Correctional facilities − Online interventions	3–24 months postintervention	12%–68%	− Reduction in IPV − Changes in aggressive behaviors and attitudes toward gender norms − Improvements in parenting behaviors and emotional regulation − Reduction in substance use (alcohol, drugs)	− Self‐reports by fathers − Partner reports − Police records and official documentation − Parent–child interaction assessment	− Father‐focused interventions showed an overall reduction in IPV behaviors − Programs that integrated gender‐transformative approaches had the strongest impact on IPV reduction − CBT‐based and Motivational Interviewing programs were the most effective for reducing aggression and improving emotional regulation

*Note:* *ACT: Acceptance and Commitment Therapy; *ASMT: Anger Self‐Management Training; *BAI: Brief Alcohol Intervention; *BCT: Behavioral Couples Therapy; *BIP: Batterer Intervention Program; *BT: Behavioral Therapy; *CAT: Cognitive Analytic Therapy; *CBT: Cognitive‐Behavioral Therapy; *CCP: Couple Care for Parents of Newborns; *DBT: Dialectical Behavior Therapy; *DV: Domestic Violence; *F4C: Fathers for Change; *FORNET: Forensic Offender Rehabilitation Narrative Exposure Therapy; *GATHER: Greeting, Ask, Tell, Help, Explain, and Refer; *IBM‐H: Interpretation Bias Modification‐Hostility training; *IBT: Individually Based Treatment; *IMP: Individualized Motivational Plan; *IPV: Intimate Partner Violence; *ME: Motivational Enhancement Therapy; *MI: Motivational Interviewing; *REAL: Responsible, Engaged, and Loving Fathers Initiative; *RNR: Risk‐Need‐Responsivity Model; *SAH‐C: Strength at Home‐Couples Program; *SAH‐M: Strength at Home‐Men's Program; *SADV: Standardized Aggression and Domestic Violence Treatment; *SBI: Standard Batterer Intervention; *SBP: Standard Batterer Program; *SOCMI: Stages of Change Motivational Interviewing; *SU: Substance Use; *SU/SA: Substance Use/Substance Abuse; *SU Treatment: Substance Use Treatment; **ANSIE: The Nowicki‐Strickland Internal External Locus of Control; **ASES: The Adult Self‐Expression Scale; **AUDIT: Alcohol Use Disorders Identification Test; **CTS: Conflict Tactics Scale; **CTS‐R/CTS‐2: Conflict Tactics Scale Revised; **DAS(1): Danger Assessment Scale; **DAS (2): Dyadic Adjustment Scale; **DDQ: Daily Drinking Questionnaire; **EAQ: The Emotional Abuse Scale; **GCS: The Generalised Contentment Scale; **ICS: The Index of Clinical Stress; **IDI: The Interpersonal Dependency Inventory; **IFS: The Index of Family Relations; **IMS: The Index of Marital Satisfaction; **IRI: Interpersonal Reactivity Index; **MMEA: Multidimensional Measure of Emotional Abuse; **MCTS: Modified Conflict Tactics Scale; **OAS: Overt Aggression Scale; **PAS: The Personality Assessment Screener; **PASPH: Partner Abuse Scale–Physical; **PCQ: The Processes of Change Scale; **PDAAD: Percentage of Days Abstinent from Alcohol; **PHDD: Percentage Heavy Drinking Days; **RSE: The Rosenberg Self‐Esteem Index; **SRIS: The Sex‐Role Ideology Scale Short Form; **TLFB: Time Line Follow Back; **TSC‐40: The Trauma Symptom Checklist‐40; **URICA: The University of Rhode Island Change Assessment; **URICA‐DV: The University of Rhode Island Change Assessment–Domestic Violence; **WAI: Working Alliance Inventory.

#### 3.1.5 Type of Intervention

The systematic reviews reveal substantial heterogeneity in intervention strategies, which can be broadly grouped into punitive‐judicial and rehabilitative psychoeducational or therapeutic approaches. Justice‐based interventions—including arrest policies, prosecution, specialized domestic violence courts, and protection orders—aim primarily to deter future violence through legal accountability, and the immediate containment of harm. These strategies operate through mechanisms that differ markedly from those of therapeutic interventions, which seek to reduce risk by fostering behavioral change and draw on established psychological and criminological frameworks, such as the Risk‐Need‐Responsivity (RNR) model (Andrews et al. 2006), widely supported within the violence prevention literature.

Rehabilitative interventions can be further divided into traditional programs, which rely on standardized “one‐size‐fits‐all” formats, and alternative or next‐generation models that tailor treatment to perpetrators' co‐occurring characteristics and criminogenic needs (e.g., substance use, trauma histories, emotional dysregulation, relational patterns). These approaches increasingly adopt individualized treatment plans or integrated individual and couple‐based modalities to enhance their relevance and effectiveness.

Two reviews (Benitez et al. 2010; Stjernqvist and Strand 2024) focused exclusively on judicial measures such as arrest, protection orders, and IPV‐specific courts. The majority of systematic studies, however, examined the effectiveness of psychoeducational and therapeutic models, many of which encompassed both traditional and alternative paradigms. Within this group, two systematic reviews analyzed interventions implemented solely in European contexts (Akoensi et al. 2013; Lilley‐Walker et al. 2018), while one focused specifically on studies conducted in Spain (Ferrer‐Perez and Bosch‐Fiol 2018). Additionally, two reviews explicitly compared outcomes across traditional versus individualized intervention models (Eckhardt et al. 2013; Waller 2016).

A smaller subset of reviews addressed specific intervention modalities. For instance, one review assessed the effectiveness of group‐based cognitive behavioral therapy (CBT) for IPV perpetrators (Nesset et al. 2019), while another evaluated CBT with anger management components in perpetrators with co‐occurring alcohol abuse (Gilchrist et al. 2015).Focused on specialized alcohol abuse interventions for IPV offenders. Furthermore, one systematic review (Wynter et al. 2025) targeted interventions aimed specifically at father‐perpetrators to reduce recidivism. Three additional reviews (McMurran 2009; Soleymani et al. 2018; Roldán‐Pardo et al. 2024) investigated the role of motivational strategies in enhancing treatment engagement and adherence. Lastly, only one review exclusively analyzed interventions delivered in healthcare settings (Tarzia et al. 2020).

#### 3.1.6 Outcomes

Across the 23 systematic reviews included in this umbrella review, IPV recidivism was assessed in 20 studies, making it the most frequently investigated outcome. The majority of the included systematic reviews investigated the effectiveness of interventions for IPV perpetrators in reducing physically, psychological and verbal forms of violent recidivism, some of which also focused on psychological (Ferrer‐Perez and Bosch‐Fiol 2018; Nesset et al. 2019; Stith et al. 2004; Waller 2016) or verbal aggression (Akoensi et al. 2013), whereas others assessed combined IPV outcomes that included physical, psychological, and verbal forms of violence (Sousa et al. 2024; Tarzia et al. 2020).

Regarding IPV related behaviors, eight studies examined the impact of interventions on alcohol and/or substance abuse (Gilchrist et al. 2015; Pinto e Silva et al. 2023; Sousa et al. 2024; Stover et al. 2009; Tarzia et al. 2020; Vall et al. 2024) and eight more also evaluated psychological and psychopathological variables for IPV perpetrators (Ferrer‐Perez and Bosch‐Fiol 2018; Laskey 2016; Lilley‐Walker et al. 2018; Nesset et al. 2019; Roldán‐Pardo et al. 2024; Satyen et al. 2022; Sousa et al. 2024; Travaini et al. 2024). Improvements in attitude, behaviors and propensity toward IPV and violence were assessed in six studies (Ferrer‐Perez and Bosch‐Fiol 2018; Lilley‐Walker et al. 2018; Soleymani et al. 2018; Sousa et al. 2024; Stith et al. 2004; Travaini et al. 2024) while improvements in anger expression and emotional control were assessed three studies (Pinto e Silva et al. 2023; Vall et al. 2024; Wynter et al. 2025). Normative beliefs about gender norms and gender‐related attitude were assessed in two studies (Satyen et al. 2022; Wynter et al. 2025). Two reviews considered the reduction in stalking behavior on IPV perpetrators (Benitez et al. 2010; Travaini et al. 2024).

About less frequently reported but notable outcomes, one study specifically focused on father‐based primary studies included improvements in parenting skills and behaviors as one of the main outcomes (Wynter et al. 2025), another one included offenders satisfaction with treatment (Lilley‐Walker et al. 2018) and a single study assessed victim's perception of safety (Akoensi et al. 2013).

Protection order violations were considered in one study (Benitez et al. 2010), while police involvement and new arrests rates were outcomes in five studies (Eckhardt et al. 2013; Sousa et al. 2024; Stjernqvist and Strand 2024; Travaini et al. 2024; Waller 2016).

##### 3.1.6.1 Outcome Measurement Sources

As sources to assess outcomes, official or police records (e.g., rearrest rates) were used in 16 studies, while self‐reports were used in 17, indicating that both methods were employed with comparable frequency in primary studies. Two of the included studies comprehend biological testing such as breath samples and urine toxicology tests (Sousa et al. 2024).

A various amount of validated psychometric tools was typically used to assess the said outcomes and Conflict Tactics Scale (CTS) was the most used, in 7 out of 23 studies (Gilchrist et al. 2015; Laskey 2016; Nesset et al. 2019; Soleymani et al. 2018; Stover et al. 2009; Tarzia et al. 2020). Only one study included therapists' assessment as source (Pinto e Silva et al. 2023) and a single review combined self‐reports assessment with a qualitative interview (Stith et al. 2004).

#### 3.1.7 Intervention Effectiveness

The systematic reviews included in this umbrella review generally reveal limited evidence for the effectiveness of interventions targeting IPV perpetrators, alongside consistent concerns regarding the methodological quality of the primary studies. A recurring challenge within the literature is the methodological fragility of these studies, characterized by small sample sizes, high attrition rates (often exceeding 90%), brief follow‐up periods, and the use of heterogeneous and inconsistent outcome measures. These limitations significantly undermine the robustness, replicability, and generalizability of findings. Notably, the apparent effectiveness of certain interventions tends to diminish when evaluated using more rigorous designs and when recidivism is measured through partner‐reported data rather than official records.

Despite these challenges, some of the included systematic reviews identified modest but noteworthy improvements associated with specific intervention components or contexts. Integrated or combined interventions addressing both IPV and substance use showed reductions in aggressive behavior when substance use decreased concurrently (Stover et al. 2009; Sousa et al. 2024).

Couple‐based programs demonstrated positive preliminary results in low‐to‐moderate risk samples or when alcohol/substance use was a contributing factor (Stith et al. 2004; Stover et al. 2009). Culturally adapted or gender‐transformative interventions also showed promising outcomes, including reductions in abuse episodes and improvements in communication and gender‐related attitudes (Satyen et al. 2022; Wynter et al. 2025).

Motivational Interviewing (MI)—both exclusive or combined with CBT—consistently improved treatment engagement, readiness for change, and program completion (Eckhardt et al. 2013; Pinto e Silva et al. 2023; Roldán‐Pardo et al. 2024; Soleymani et al. 2018), although its effects on IPV behaviors were mixed or non‐significant across most reviews (McMurran 2009; Soleymani et al. 2018). Interventions targeting emotion regulation, such as DBT, or ACT led to improvements in anger management and impulse control (Travaini et al. 2024), but these psychological gains did not systematically translate into consistent reductions in IPV recidivism.

Traditional Duluth‐based models and standard group CBT programs generally presented weak or inconsistent evidence of effectiveness (Akoensi et al. 2013; Lilley‐Walker et al. 2018; Vall et al. 2024), while CBT combined with MI showed promising effects on reducing aggression, recidivism and improving emotional regulation in two different reviews (Vall et al. 2024; Wynter et al. 2025).

A detailed summary of population characteristics, intervention modalities, and the principal outcomes of the systematic reviews is presented in Table 4.

### 3.2 Meta‐Analysis

#### 3.2.1 Characteristics of the Studies

**Figure 3:**
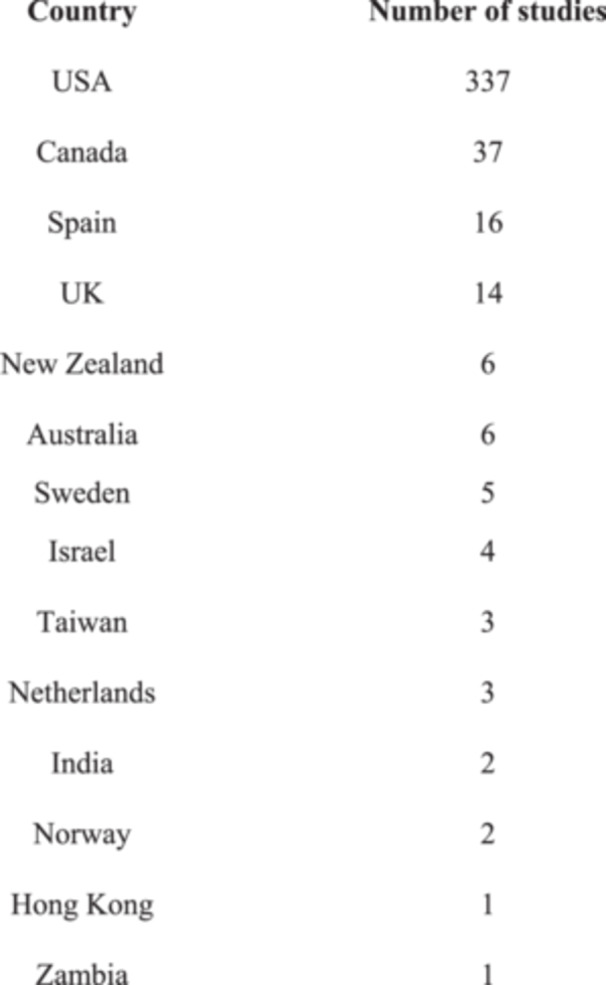
Geographic origin of primary studies (meta‐analyses).

The 18 meta‐analysis were published in scientific journals between 2004 and 2024. They include 437 primary studies conducted between 1988 and 2022 in diverse geographical regions, predominantly conducted in the United States (77%), Canada (8,5%), Spain (3,6%), and the United Kingdom (3%) (See Figure 3). The reviews were conducted in the United States (8), Spain (3), the United Kingdom (1), Norway (1), or in collaboration with multiple countries (5), including Denmark, Turkey, Canada, and Australia among others.

Most of the meta‐analysis investigated the effectiveness of interventions targeting IPV perpetrators in reducing violent, psychological, and verbal behavior (recidivism). A few studies also examined the effectiveness of interventions directed at variables correlated with IPV perpetration, such as protection orders violation (Cordier et al. 2021) and substance abuse (Santirso et al. 2020; Stephens‐Lewis et al. 2021). These studies explored the moderating effect of such variables on IPV perpetration.

Within each meta‐analysis, there are between 6 and 70 primary studies, and the total sample size of the meta‐analysis is of 328,251 participants.

Sample size data were not available for two of the included meta‐analysis (Babcock et al. 2004; Feder and Wilson 2005).

#### 3.2.2 Research Designs

Regarding the research design, most of the meta‐analyses included both randomized clinical trials (RCTs) and quasi‐experimental primary studies (Babcock et al. 2024; Cheng et al. 2021; Feder and Wilson 2005; Fernández‐Fernández et al. 2022; Gannon et al. 2019; Wilson et al. 2021) and five out of the eighteen meta‐analysis, included in this umbrella review, presented exclusively RCTs (Karakurt et al. 2016; Oğuztüzün et al. 2023; Santirso et al. 2020; Smedslund et al. 2007; Stephens‐Lewis et al. 2021).

Four meta‐analyses included experimental and quasi‐experimental studies (Arce et al. 2020; Arias et al. 2013; Babcock et al. 2004; Garner et al. 2021), while only one of the studies presented case series, case controls, cohort retrospective and cohort prospective reports (Cordier et al. 2021), another one combined pre‐post comparison studies and RCTs (Karakurt et al. 2019), one presented RCTs, quasi‐experimental combined with RCTs 2 active treatment and quasi‐experimental 2 active treatment (Travers et al. 2021).

#### 3.2.3 Quality Assessment of Meta‐Analyses

Table 2 presents the quality assessment of the included systematic reviews, detailing individual items and overall ratings. Among the meta‐analyses, seven reviews were rated as having high overall reliability (Smedslund et al. 2007; Feder and Wilson 2005; Karakurt et al. 2016; Karakurt et al. 2019; Cordier et al. 2021; Stephens‐Lewis et al. 2021; Wilson et al. 2021), while six received a low rating (Babcock et al. 2004; Arias et al. 2013; Arce et al. 2020; Cheng et al. 2021; Fernández‐Fernández et al. 2022; Garner et al. 2021). Four meta‐analyses were rated as moderate in reliability (Gannon et al. 2019; Santirso et al. 2020; Travers et al. 2021; Babcock et al. 2024), and one was deemed critically low (Oğuztüzün et al. 2023), reflecting considerable heterogeneity in methodological rigor across the literature.

In summary, many meta‐analyses exhibited methodological shortcomings, including the absence of comprehensive search strategies, failure to report the use of duplicate procedures for study selection and data extraction, and inconsistent or insufficient assessment of risk of bias. Furthermore, risk of bias evaluations were often not incorporated into the interpretation of results, thereby undermining the transparency, reliability, and overall strength of the evidence synthesis.

#### 3.2.4 Sample Characteristics

Across the meta‐analyses, the population is highly heterogeneous, except for the gender of the participants. The samples mainly consist of male individuals, 6 out of 18 meta‐analyses only included primary studies that presented exclusively male subjects (Cheng et al. 2021; Feder and Wilson 2005; Karakurt et al. 2019; Smedslund et al. 2007; Stephens‐Lewis et al. 2021; D. B. Wilson et al. 2021) while one study included primary studies with generally balanced gender samples, comprising 50% male and 50% female participants (Karakurt et al. 2016). Nonetheless, it is important to highlight that the majority of studies (11) did not specify the percentage of male participants (Table 5) and besides percentage we know that four meta‐analyses included both male and female perpetrators (Babcock et al. 2024; Cordier et al. 2021; Santirso et al. 2020; Travers et al. 2021).

**Table 5:** Features meta‐analyses.

				Population					
References	Name of journal	Country	N. of primary studies	N. of subject	Gender (% maschi)	Age	Research design	Type of intervention*	Overall confidence (AMSTAR)
Arce et al. (2020)	*Psychosocial Intervention*	Spain	25	20,860	nr	nr	Experimental quasi‐experimental	Duluth CBT CBT individual RNR model Psychodynamic Mindfulness	Low
Arias et al. (2013)	*Psychosocial Intervention*	Spain	19	18,941	nr	nr	Experimental quasi‐experimental	Duluth CBT Duluth + CBT Psychodynamic Mindfulness	Low
Babcock et al. (2004)	*Clinical Psychology Review*	United States	22	nr	nr	nr	Experimental quasi‐experimental	Duluth/femminist/psychoeducational CBT Couples therapy Supportive therapy	Low
Babcock et al. (2024)	*Clinical psychology review*	United States	59	20,730	nr	nr	RCT Quasi‐experimental	Duluth CBT Other (multicouple group interventions, Acceptance and Commitment Therapy (ACT) groups, and restorative justice interventions)	Moderate
Cheng et al. (2021)	*Trauma, Violence & Abuse*	United States	17	4579	100	nr	RCT quasi‐experimental	Duluth psychoeducational CBT Olistic RNR model	Low
Cordier et al. (2021)	*Trauma, Violence & Abuse*	Australia Norway	25	31,586	nr	> 17	Case series case control cohort retrospective cohort prospective	Protection orders	High
Feder et al. (2008)	*Campbell Systematic Reviews*	United States	10	nr	100	nr	RCT quasi‐experimental	CBT psychoeducational psychoeducational + CBT (all court‐ordered, almost all in probation)	High
Fernández‐Fernández et al. (2022)	*Anuario de Psicologìa Jurìdica*	Spain	26	19,773	nr	nr	RCT quasi‐experimental	Duluth/psychoeducational CBT Anger management Psychodynamic RNR model mindfulness ACT	Low
Gannon et al. (2019)	*Clinical Psychology Review*	United Kingdom Canada	70	55,000	nr	nr	RCT quasi‐experimental	CBT Duluth Psychoeducational	Moderate
Garner et al. (2021)	*Journal of Criminal Law and Criminology*	United States	57	127,237	nr	nr	Experimental quasi‐experimental	Postarrest sanctions (prosecution, conviction, incarceration)	Low
Karakurt et al. (2016)	*Journal of Marital and Family Therapy*	United States	6	470	50	34	RCT	Individual couple therapy conjoint group therapy combination of both	High
Karakurt et al. (2019)	*Neuroscience and Biobehavioral Reviews*	United States Turkey	17	1492	100	31.5−46.4	Pre‐post comparison pre‐post RCT	Duluth + CBT CBT CBT + SU CBT/psychodynamic/femminist ME therapy CBT + MI MI	High
Oğuztüzün et al. (2023)	*Psychosocial Intervention*	United States	16	703	nr	nr	RCT	CBT SBI SADV SUB Sex Roles Psychodynamic + Attachment + Feminist MI IMP	Critically low
Santirso et al. (2020)	*Psychosocial Intervention*	Spain United Kingdom	12	1733	82,7	33.47	RCT	Motivational strategies (for IPV/SU/couples) Before or after IPV treatment	Moderate
Smedslund et al. (2007)	*Cochrane Database of Systematic Reviews*	Norway	6	3204	100	31.9 (only 3 studies)	RCT	CBT Intervention with CBT components	High
Stephens‐Lewis et al. (2021)	*Trauma, Violence & Abuse*	United Kingdom	9	1014	100	36.2	RCT	IPV + SU CBT + SU CBT + MI + SU MI + SU MI	High
Travers et al. (2021)	*Clinical Psychology Review*	Denmark United Kingdom	31	19,309	97,5	34.2	RCT quasi‐sperimentale RCT 2 active treatment quasi‐experimental 2 active treatment	Duluth CBT CBT7Duluth/femminist CBT/psychodynamic SOMCI MI CBT + MI CBT + SU Alcohol abuse intervention mindfulness Group couple's therapy CBT for veterans (trauma‐informed)) RNR model	Moderate
Wilson et al. (2021)	*Campbell Systematic Reviews*	United States	11	4824	100	nr	RCT quasi‐experimental	Duluth CBT Duluth + CBT Psychoeducational Judicial monitoring Rigorous monitoring with counseling SU (all court‐mandated, almost all in probation)	High

*Note:* *ACT: Acceptance and Commitment Therapy; ASMT: Anger Self‐Management Training; BAI: Brief Alcohol Intervention; BCT: Behavioral Couples Therapy; BIP: Batterer Intervention Program; BT: Behavioral Therapy; CAT: Cognitive Analytic Therapy; CBT: Cognitive‐behavioral therapy; CCP: Couple Care for Parents of Newborns; CTS: Conflict Tactics Scale; DBT: Dialectical Behavior Therapy; F4C: Fathers for Change; FORNET: Forensic Offender Rehabilitation Narrative Exposure Therapy; GATHER: Greeting, Ask, Tell, Help, Explain, and Refer; IBM‐H: Interpretation Bias Modification‐Hostility training; IBT: Individually Based Treatment; IMP: Individualized Motivational Plan; IPV: Intimate Partner Violence; ME: Motivational Enhancement Therapy; MI: Motivational Interviewing; REAL: Responsible, Engaged, and Loving Fathers Initiative; RNR: Risk‐Need‐Responsivity Model; SAH‐C: Strength at Home‐Couples Program; SAH‐M: Strength at Home‐Men's Program; SADV: Standardized Aggression and Domestic Violence Treatment; SBI: Standard Batterer Intervention; SBP: Standard Batterer Program; SOCMI: Stages of Change Motivational Interviewing; SU: Substance Use; SU Treatment.

In addition to gender, the sample characteristics reveal considerable diversity across meta‐analyses. Four studies included interventions delivered to couples rather than individual perpetrators (Babcock et al. 2004; Cheng et al. 2021; Cordier et al. 2021; Travers et al. 2021) while two specifically included only heterosexual male subjects (Stephens‐Lewis et al. 2021; D. B. Wilson et al. 2021). Regarding legal status, six meta‐analyses incorporated participants who were court‐mandated to attend intervention programs (Feder and Wilson 2005; Karakurt et al. 2019; Santirso et al. 2020; Smedslund et al. 2007; Travers et al. 2021; Wilson et al. 2021), and four of these also included voluntary participants (Feder and Wilson 2005; Karakurt et al. 2019; Santirso et al. 2020; Smedslund et al. 2007). Four reviews focused on individuals with substance use disorders (Santirso et al. 2020; Smedslund et al. 2007; Stephens‐Lewis et al. 2021; Travers et al. 2021), while another four included military populations (Cheng et al. 2021; Feder and Wilson 2005; Smedslund et al. 2007; Wilson et al. 2021). Additionally, three meta‐analyses explicitly excluded participants with intellectual disabilities or severe mental disorders (Gannon et al. 2019; Stephens‐Lewis et al. 2021; Travers et al. 2021).

#### 3.2.5 Type of Intervention

Regarding the types of interventions, the included meta‐analyses report a considerable heterogeneity in the approaches adopted across primary studies. Cognitive‐behavioral therapy (CBT) emerged as the most frequently employed model, either in its standard form or integrated with other components such as substance use (SU) treatment or motivational interviewing (MI), and was reported in 14 out of 18 meta‐analyses. The Duluth model or feminist‐psychoeducational frameworks were also widely used and documented in 10 reviews (Arce et al. 2020; Arias et al. 2013; Babcock et al. 2004; Babcock et al. 2024; Cheng et al. 2021; Fernández‐Fernández et al. 2022; Gannon et al. 2019; Karakurt et al. 2019; Travers et al. 2021; Wilson et al. 2021). Several reviews also included third‐wave cognitive‐behavioral approaches, such as Acceptance and Commitment Therapy (ACT) (Babcock et al. 2024) or mindfulness‐based interventions (Arce et al. 2020; Arias et al. 2013; Fernández‐Fernández et al. 2022; Travers et al. 2021).

Furthermore, couple‐based treatments (e.g., individual group therapy, group couples therapy, multi‐couple therapy) were explored in five meta‐analyses (Babcock et al. 2004; Babcock et al. 2024; Karakurt et al. 2016; Santirso et al. 2020; Travers et al. 2021).

Among the studies, some meta‐analysis specifically focused on a single intervention modality: one meta‐analysis included only protection orders as the intervention strategy (Cordier et al. 2021), while another focused exclusively on the effectiveness of postarrest sanctions, including prosecution, conviction, and incarceration (Garner et al. 2021). A further study investigated the use of motivational strategies applied before or after IPV treatment to enhance engagement and readiness to change (Santirso et al. 2020). Lastly, one meta‐analysis examined solely Cognitive‐Behavioral Therapy (CBT) and interventions incorporating CBT components (Smedslund et al. 2007).

#### 3.2.6 Outcomes

Across the 18 meta‐analyses included in this umbrella review, IPV recidivism was assessed in 15 studies, making it the most frequently investigated outcome. A total of three studies (Karakurt et al. 2016; Karakurt et al. 2019; Oğuztüzün et al. 2023) reported quantitative reductions in violence, including reductions in total, physical, psychological, and emotional IPV, or changes in the heterogeneity and severity of violent behavior. Protection order violations were considered in one study (Cordier et al. 2021), while police involvement and new arrests rates were outcomes in 5 studies (Feder and Wilson 2005; Garner et al. 2021; Smedslund et al. 2007; Travers et al. 2021; Wilson et al. 2021). Beyond violence reduction and recidivism, substance use (SU) was investigated in three studies (Santirso et al. 2020; Smedslund et al. 2007, 2011; Stephens‐Lewis et al. 2021), using both self‐report instruments (e.g., Time Line Follow Back, Addiction Severity Index, Substance Use Calendar) and biological testing (breath samples, urine toxicology). Similarly, normative beliefs about IPV and alcohol were assessed in two studies (Santirso et al. 2020; Stephen‐Lewis et al. 2021) and drop‐out intervention dose in one study (Santirso et al. 2020).

About less frequently reported but notable outcomes, one meta‐analysis included victim satisfaction with the justice system, perpetrators' attitudes, beliefs, and behavioral changes (Smedslund et al. 2007), another one included, among the other outcomes, marital satisfaction, blame attribution and partners and children's mental health (Stephens‐Lewis et al. 2021) and, lastly, a single study assessed the partner perception of safety as an outcome (Travers et al. 2021).

##### 3.2.6.1 Outcome Measurement Sources

These outcomes were frequently assessed through official or police records (e.g., arrest, charge, conviction) and complemented by partner reports in 13 studies (Babcock et al. 2004; Babcock et al. 2024; Cheng et al. 2021; Feder and Wilson 2005; Fernández‐Fernández et al. 2022; Garner et al. 2021; Karakurt et al. 2016; Karakurt et al. 2019; Santirso et al. 2020; Smedslund et al. 2007; Stephen‐Lewis et al. 2021; Travers et al. 2021; Wilson et al. 2021) and self‐reports in 7 studies (Cheng et al. 2021; Karakurt et al. 2016; Santirso et al. 2020; Smedslund et al. 2007; Stephens‐Lewis et al. 2021; Travers et al. 2021; Wilson et al. 2021).

These were typically evaluated through self and partner reports and validated psychometric tools such as the Conflict Tactics Scale (CTS), used in 4 out of 18 meta‐analyses (Oğuztüzün et al. 2023; Smedslund et al. 2007; Stephens‐Lewis et al. 2021; Travers et al. 2021) in one case, combined with a qualitative interview (Smedslund et al. 2007).

#### 3.2.7 Intervention Effectiveness

The findings of the included meta‐analyses reflect a generally uncertain and inconsistent landscape regarding the effectiveness of interventions targeting IPV perpetrators. Overall, detected effect sizes tend to be modest and frequently nonsignificant, with values ranging from small to moderate. However, wide confidence intervals—often encompassing zero—underscore the high degree of statistical uncertainty. A consistent pattern emerges whereby effect sizes diminish in studies employing more rigorous designs, such as randomized controlled trials (RCTs), and appear more pronounced when recidivism is assessed via official records as opposed to partner reports, which tend to yield null or inconsistent findings (Babcock et al. 2004; Babcock et al. 2024; Cheng et al. 2021; Wilson et al. 2021).

Despite this general trend, several reviews point to encouraging outcomes. For instance, Fernández‐Fernández et al. (2022) report moderate and significant effects for cognitive‐behavioral therapy (CBT; δ = 0.57), alternative interventions (δ = 0.59), and the Duluth model (δ = 0.51), particularly when programs exceed 12 weeks in duration or when follow‐up extends beyond 1 year. Gannon et al. (2019) identify a significant reduction in recidivism (OR = 0.65), while Travers et al. (2021) observe a decreased risk among participants in programs aligned with Risk‐Need‐Responsivity (RNR) principles, although effectiveness declines over time. Cordier et al. (2021) highlight the utility of protective interventions (e.g., restraining orders), though such strategies may not produce direct behavioral changes in perpetrators. Nevertheless, the heterogeneity of studies and the diversity of intervention models—including CBT, Duluth, psychodynamic therapy, mindfulness‐based approaches, motivational interviewing (MI), and acceptance and commitment therapy (ACT)—pose significant challenges in drawing definitive conclusions about their comparative effectiveness.

In sum, while certain modalities such as CBT, RNR‐aligned interventions, and motivation‐enhancing strategies show promise, the overall evidence base suggests that intervention effectiveness remains limited and is significantly influenced by methodological quality and contextual factors.

Descriptive characteristics of the study populations, intervention types, and the main findings of the meta‐analyses are detailed in Table 6.

**Table 6:** Meta‐analyses.

		Treatment					Outcome		Main results
References	Feautures of the population	Model of intervention*	Duration	Setting	Follow‐up	Attrition rate (%)	Measure*	Source**	Effect size with 95% CI, p, I²
Arce et al. (2020)	IPV perpetrators	Duluth CBT CBT individual RNR model Psychodynamic Mindfulness	5 days − 36 weeks 8–78 sessions	− Group − Individual − Couple − Couple group	6 months – 10 years	nr	IPV recidivism	− Official record − Self‐partner report	− General: δ = 0.44, 80% CI [−0.13, 1.00]
Arias et al. (2013)	IPV perpetrators	Duluth CBT Duluth + CBT Psychodynamic Mindfulness	5 days − 36 weeks	− Group − Individual − Couple − Couple group	6 months ‐ 10 years	nr	IPV recidivism	− Official record − Self‐partner report	− General: δ = 0.42, 90% CI [−0.07, 0.91]
Babcock et al. (2004)	IPV perpetrators couples	Duluth/femminist/psychoeducational CBT Couples therapy Supportive therapy	< 16 weeks > 16 weeks	Group	< 12 months > 12 months	18–84	IPV recidivism	− Police record − Partner report	− Police report: *d* = 0.18, 95% CI [0.11–0.25], *p* < 0.5 − Partner report: *d* = 0.18, 95% CI [0.08–0.28]; *p* < 0.05
Babcock et al. (2024)	IPV perpetrators (men and women)	Duluth CBT Other (multicouple group interventions, Acceptance and Commitment Therapy (ACT) groups, and restorative justice interventions)	8–52 weeks	Community setting Court‐mandated programs	3 months to 11 years	From 10% to 70%	‐ Recidivism rates	− Police reports − Partner reports	Police report: g = 0.29, 95% CI [0.20, 0.38], I² = 81.4% Partner report: g = 0.21, 95% CI [0.02, 0.41], I² = 84.1%
Cheng et al. (2021)	IPV perpetrators − Convicted − Nonconvicted couples − US Navy military	Duluth psychoeducational CBT Olistic RNR model	nr	Group	6 months ‐ 10 years	nr	− IPV recidivism − Official recidivism	− Official record − Self‐report − Partner report − CTS‐R	− Police report in quasi‐experimental: OR: 0.15, 95% CI [0.06, 0.37], *p* < 0.001 − Official record in RCT: OR: 0.74, 95% CI [0.49, 1.10]; *p* = 0.140‐partner‐report in RCT: OR = 0.82, 95% CI [0.57, 1.19] *p* = 0.296
Cordier et al. (2021)	IPV perpetrators − Male − Male and female Couples	Protection orders	nr	nr	40 days ‐ 4 years	nr	Protection orders violation	− Partner report − Police record	− Police report: OR: 0.353, 95% CI [0.298, 0.412], *p* < 0.001 − Restriction order (no arrest) OR: 0.265, 95% CI [0.224, 0.310], *p* < 0.001
Feder et al. (2008)	IPV perpetrators − Heterosexual − Court‐mandated − Court‐mandated and voluntary − Probation − U.S. Navy military	CBT psychoeducational psychoeducational + CBT	8−32 sessions	Group	Minimum 6 months	nr − alto tasso di abbandono partner report	− IPV recidivism − Official IPV recidivism (new arrest, charge, conviction)	− Official record − Partner report CTS/CTS‐2	− Partner report: d = −0.00, 95% CI [−0.12, 0.11 − Report polizia: d = 0.26, 95% CI [0.03, 0.50]; *p* < 0.5
Fernández‐Fernández et al. (2022)	IPV perpetrators	Duluth/psychoeducational CBT Anger management Psychodynamic RNR model mindfulness ACT	< 16 weeks > 16 weeks	nr	< 12 months >/= 12 months	nr	IPV recidivism	− Official record − Partner report (1 study)	− General: δ:0.54, 90% CI [−0.18 0.90], Sδw2 = 0.049, *p* < 0.001 − Intervention < 16 weeks: δw = 0.39, 90% CIδ [0.09, 0.87] − Follow‐up >/=12 months: δw = 0.38, 90% CI [0.14, 0.90] − Type of intervention: CBT: δw = 0.57; 90% CI [.04, 1.18] alternative: δw = 0.59, 90% CIδ [−0.18, 1] Duluth: δw = 0.51, 90% CI δ [−0.23, 0.79]
Gannon et al. (2019)	IPV perpetrators without: Learning disability or other cognitive impairment, or committed to a mental health facility due to a significant mental disorder	CBT Duluth Psychoeducational	100–200 h	− Group − Closed group − Community‐based	62 months (on average)	nr	IPV recidivism	nr	− General: OR = 0.65, 95% CI = 0.44, 0.97; Qs = 72.84 *p* < 0.001
Garner et al. (2021)	IPV perpetrators alleged/charged/convicted for IPV offense	Postarrest sanctions (prosecution, conviction, incarceration)	nr	nr	6 months ‐ more than 5 years	nr	− IPV recidivism − Official IPV recidivism (new arrest, charge, conviction)	− Partner report − Official record	− Prosecution: OR: −0.196, 95% CI [−0.43 – 0.03], *p* = 0.095 − Conviction: OR: 0.21, 95% CI [−0.20 – 0.24], *p* = 0.85 − Incarceration: OR: 0.367, 95% CI [0.15 −0.59], *p* < 0.001
Karakurt et al. (2016)	IPV Perpetrators	Behavioral Couple Therapy (BCT), Cognitive‐Behavioral Couple Therapy (CBCT), Integrative BCT (IBCT)	8–20 sessions	Community setting Court‐mandated setting	nr	nr	Reductions in male—and female—perpetrated violence	Self‐reports Partner reports Official records	WMD = −0.84; 95% CI [−1.37, −0.30]; *p* = 0.00; I² = 0%
Karakurt et al. (2019)	IPV perpetrators − Court‐mandated − Voluntary	Duluth + CBT CBT CBT + SU CBT/psychodynamic/feminist ME therapy CBT + MI MI	12– 70 h	− Group − Individual telephone delivered	End of treatment ‐ 6 months	3–41 (in media > 24)	− Reduction in total violence − Reduction in severity of violence	− Partner report − Official record	− General: OR: −0.85; 95% CI [−1.02 to −0.69]
Oğuztüzün et al. (2023)	IPV perpetrators	Cognitive Behavioral Therapy (CBT) Standard Batterer Intervention (SBI) Standard Violence Treatment (SADV) Substance Abuse Treatment (SUB) Sex Roles Psychodynamic + Attachment + Feminist Motivational Treatment (MI) Individualized Motivational Plan (IMP)	nr	nr	nr	nr	− Relative reduction in violence − Change in heterogeneity of violence in the study sample	Conflict Tactics Scale (CTS)	SMD ≈ −0.40, 95% CI = n.r., *p* < 0.05; I² = n.r. (VF used)
Santirso et al. (2020).	IPV perpetrators − Court‐mandated − Voluntary − Male − Male and female − SUD	Motivational strategies (for IPV/SU/couples) Before or after IPV treatment	1–41 sessions	− Group − Individual − Couple − Community‐based DV agency ‐Family center clinic − University ‐Community +phone/mail	End of treatment ‐ 12 months	riportato come misura di outcome	− Official recidivism − IPV recidivism − Physical IPV − Psychological IPV − Emotional abuse − Drop‐out and intervention dose − SU − Perceived norms on IPV/drinking	− Self‐report − Partner report − Official record − CTS/MCTS/ CTS‐2 − MMEA − TLFB‐AM	− Reduction of attrition rate: OR = 1.73, 95% CI [1.04, 2.89]; I² = 0% − Intervention dose: SMD = 0.27, 95% CI [0.08, 0.45]; I² = 0% − IPV official records: OR = 1.46, 95% CI [0.76, 2.80]; I² = 33%‐physical IPV self‐report: 0.09, 95% CI [−0.21, 0.38]; I² = 0% − Psychological IPV self‐report: 0.09, 95% CI [−0.21, 0.38]; I² = 53%
Smedslund et al. (2007)	Physical IPV perpetrators − Court‐mandated − Voluntary − Military − SUD	CBT Intervention with CBT components	2–12 months	Group	nr	nr	− Official recidivism (new arrest/crime) − IPV recidivism − Victim satisfaction with JS − Perpetrator's belifs, attitudes and behavior − SU (baseline)	− Official record − Partner report − Self‐report − Perpetrator/victim interview − CTS‐R/MCTS − SCID − ASI − SUC − Breath samples, urine toxicology screens	− General: RR: 0.86, 95% [0.54−1.38]
Stephens‐Lewis et al. (2021)	IPV perpetrators heterosexual males wiith SU (67%–100% del campione) without mental health diagnosis	IPV + SU CBT + SU CBT + MI + SU MI + SU MI	1 sessions ‐ 16 weeks	− Group − Individual − Group + individual − SU outpatient facility − SU inpatient facility − Community +phone − Perp community − Psychology clinic − DV agency	End of treatment ‐ 12 months	24	− Physical IPV − Perpetrator's perceived norms on IPV − SU − Marital satisfaction − Blame attribution − Partner/children mental health	− Partner + self‐report − Partner report − Self‐report − CTS/R‐CTS − ISA − TLFB − URICA − Toxicology screens	− IPV: MD: 0.1 CI [−0.37, 0.57], *p* = 0.68; I² = 51% − Substance abuse: MD = 2.07, CI [0.00, 4.13], *p* = 0.05, I² = 0%
Travers et al. (2021)	Perpetratori IPV − Male − Male and female (39,6% female) − Alcohol abuse − Court‐mandated − Senza disabilità intellettiva, diagnosi disturbo mentale, SU couples of unversity students with alcohol abuse and male violence	Duluth CBT CBT7Duluth/feminist CBT/psychodynamic SOMCI MI CBT + MI CBT + SU Alcohol abuse intervention mindfulness Group couple therapy CBT for veterans (trauma‐informed) RNR model	1–35 sessions	− Group ‐Individual ‐Group + individual ‐Couples − Community‐based ‐Clinic‐based − Prison‐based	End of treatment ‐ 5 years	20 ‐ > 50	− IPV recidivism − Official recidivism (new charge, arrest, conviction) − Police involvement − Partner perception of safety	− Self‐report − Partner report − Official record − CTS − DAS − MMEA − ISA − SVAWS	Adherence to RNR principles: − Short follow‐up: OR 0.52 95% CI 0.35–0.78., *p* < 0.001; I² = 55% − Medium follow‐up: OR 0.60 95% CI [0.46–0.78], *p* < 0.001 − Long follow‐up: OR 0.75, 95% CI [0.45–1.26], *p* = 0.28.
Wilson et al. (2021)	perpetratori IPV ‐Maleheterosexualcourt mandated − Probation − Military	Duluth CBT Duluth + CBT Psychoeducational Judicial monitoring Rigorous monitoring with counselling SU	10–32 sessions 8–52 weeks	Group	6– 12 months	33	− IPV recidivism − Police involvement − Arrest	− Self‐report + official record/partner report CTS/CTS‐2	− Partner report: OR: 0.74, 95% CI [0.47, 1.16], I² = 48% − Report polizia: OR: 0.67, 95% CI [0.43, 1.05]; I² = 44%

*Note:* *IPV: intimate partner violence; CBT: Cognitive‐behavioral therapy; SOCMI: Stages of Change Motivational Interviewing MI: Motivational Interviewing ME: Motivational Enhancement Therapy; SU: Substance Use; RNR: modello Risk‐Need‐Responsivity; ACT: Acceptance and Commitment Therapy; SG: Sistema Giudiziario. ** CTS: Conflict Tactic Scale; MCTS: Modified Conflict Tactic Scale; CTS‐R/CTS‐2: Conflict Tactic Scale Revised; MMEA: Multidimensional Measure of Emotional Abuse; TLFB: Time Line Follow Back; TLFB‐AM: Time Line Follow Back‐Aggression Module; SCID: Structured Clinical Interview for DSM; ASI: Addiction Severity Index; SUC: Substance Use Calendar; URICA: The University of Rhode Island Change Assessment; ISA: Index of Spouse Abuse; DAS: Dyadic Adjustment Scale; SVAWS: Severity of Violence against Women Scale.

## 4 Discussion

Taken together, the findings of this umbrella review point to three overarching conclusions. First, both primary studies and systematic reviews exhibit substantial methodological weaknesses—such as limited study designs, inconsistent outcome measures, and high attrition—that constrain the reliability and interpretability of current evidence. Second, the effectiveness of IPV perpetrator interventions remains mixed and generally modest across program types, with positive effects often confined to specific subgroups or short‐term follow‐up periods. Third, the generalizability of findings is undermined by the predominance of research conducted in high‐income countries, particularly the United States, with a notable absence of evidence from low‐ and middle‐income contexts. These three themes structure the discussion that follows.

### 4.1 Methodological Quality

The evidence collected from the 41 systematic studies (23 systematic reviews and 18 meta‐analyses) emphasizes significant limitations in studying the effectiveness of interventions for Intimate Partner Violence (IPV) perpetrators. Despite the extensive literature available, intervention effectiveness remains difficult to establish, as the methodological weaknesses of primary studies, as highlighted by the included reviews, limit the reliability of the existing evidence. Twenty of the included reviews were rated as “low” or “critically low” in methodological quality (Table 2), with frequent issues including small sample sizes and high dropout rates (up to 96%).

The earliest systematic study among those included (Babcock et al. 2004) is highly cited in the field of IPV and remains a foundational reference, representing the first meta‐analysis on the effectiveness of intervention for IPV perpetrators. The evidence collected in the remaining 40 systematic studies shows a similar trend to the first meta‐analytical study, highlighting the fragility of the methodological framework of intervention studies and research.

Difficulties encountered in this application area stem from challenges in defining the intervention and designing the research framework (Eckhardt et al. 2006). It is unclear whether the intervention for IPV perpetrators should serve a judicial, educational, or therapeutic function, but the identified intervention strategies show an overlap of these three dimensions without pinpointing a modality that stands out in terms of effectiveness.

### 4.2 Intervention Effectiveness

Most systematic reviews and meta‐analyses related both traditional (standardized, group‐based interventions such as Cognitive‐Behavioral Therapy or Duluth‐type programs) and alternative rehabilitative interventions report a lack of significant effectiveness in reducing or preventing IPV recidivism. In the best cases, the intervention's effect is small (Babcock et al. 2004; Gannon et al. 2019) or sustained only for short to medium‐term follow‐up periods (Fernández‐Fernández et al. 2022; Travers et al. 2021; Wilson et al. 2021). Positive significant results reported through official measures (such as police reports) are not corroborated when recidivism measures through partner reports are used (Feder and Wilson 2005; Wilson et al. 2021) or when using a more rigorous research design (Cheng et al. 2021; Eckhardt et al. 2013).

Regarding traditional interventions, including standard CBT and the Duluth program, study results are conflicting or inconclusive. Two works (Smedslund et al. 2007; Nesset et al. 2019) emphasize that the evidence produced by the studies does not allow conclusions about the effectiveness of CBT intervention, while Fernández‐Fernández et al. (2022) reports a significant positive effect of such intervention compared to other modalities. Moreover, two systematic reviews show that CBT combined with MI can reveal promising effect of aggressive behaviors and recidivism reduction and improvements on IPV perpetrators emotional regulation (Vall et al. 2024; Wynter et al. 2025). The Duluth model generally demonstrates effectiveness comparable to CBT (Babcock et al. 2004) or nonsignificant effects in reducing recidivism (Fernández‐Fernández et al. 2022).

Although traditional approaches continue to dominate the field, more encouraging results emerge from next‐generation interventions that explicitly target risk factors commonly observed among IPV perpetrators. The most widely implemented of these alternative models are those addressing co‐occurring alcohol or substance misuse and those aimed at enhancing motivation for treatment. Systematic reviews consistently indicate that integrated programs combining IPV treatment with substance‐use interventions reduce aggression when decreases in alcohol or drug consumption occur simultaneously (Stover et al. 2009; Sousa et al. 2024). Meta‐analyses similarly document significant benefits for interventions jointly targeting IPV and substance misuse (Karakurt et al. 2019; Stephens‐Lewis et al. 2021). Within systematic reviews, programs incorporating anger management components show reductions in physical IPV among individuals with substance‐use problems (Gilchrist et al. 2015). Motivational approaches—including MI, ME, and SOCMI—also demonstrate significant effects in reducing dropout rates and improving treatment adherence (Eckhardt et al. 2013; Soleymani et al. 2018; Santirso et al. 2020).

Culturally adapted programs showed promising effects, particularly in relation to treatment engagement and reductions in recidivism. In the systematic review by Satyen et al. (2022) six out of ten included studies reported complete absence or marked decreases in abusive episodes.

Father‐focused interventions integrating parenting and gender‐transformative methods (Wynter et al. 2025), as well as pharmacologically integrated approaches (Travaini et al. 2024), showed notable improvements in emotional regulation, reduction in stalking or IPV‐related behaviors, and mental health outcomes. Couples‐based or integrated individual interventions show mixed results. Some reviews report non‐significant but positive effects (Stith et al. 2004; Wilson et al. 2021), whereas others indicate promising preliminary outcomes, especially among perpetrators with substance‐use problems (Stover et al. 2009; Karakurt et al. 2016).

Findings on justice‐based interventions—including arrest, prosecution, and incarceration—are likewise inconsistent. Research generally reports non‐significant or null effects for restraining orders (Benitez et al. 2010; Cordier et al. 2021) and, in some cases, adverse outcomes associated with incarceration (Garner et al. 2021). Stjernqvist and Strand (2024) similarly document mixed results: arrest may reduce recidivism in the short term but shows limited long‐term impact. It is important to note that judicial strategies differ substantially from therapeutic or psychoeducational interventions, as they aim primarily to manage immediate risk, deter future violence, and ensure victim safety rather than modify psychological mechanisms underlying perpetration. Their inclusion in the present umbrella review nonetheless reflects the heterogeneity of approaches implemented in practice and allows for a more comprehensive synthesis of available evidence.

Taken together, these findings suggest that intervention type alone does not account for treatment outcomes. Instead, programs appear more effective when they are multicomponent, tailored to individual risk profiles, and delivered with sufficient duration and engagement‐enhancing strategies. This pattern aligns with broader evidence indicating that addressing the heterogeneity of perpetrator needs is critical for achieving meaningful and sustained behavioral change.

Although generalization remains limited, systematic evidence points toward promising developments in newer intervention models and supports organizing treatment goals around variables empirically linked to perpetration. Future research should examine whether improvements in these perpetration‐related factors translate into reductions in IPV itself and should evaluate the long‐term effectiveness of emerging treatments.

### 4.3 Generalizability and Contextual Limitations

There is also a difficulty of generalizing the obtained results since the majority of primary studies were conducted in high‐income countries, particularly the United States context (61.2%) raising concerns about the generalizability of findings. Only four primary studies were conducted in low‐income countries (Rwanda, Uganda and Zambia). This consideration is particularly important because intervention in such emotionally and criminally charged field is deeply intertwined with the judicial system and the value system of the reference culture.

The implementation and research of interventions aimed at perpetrators belonging to specific populations are still lacking or entirely absent, especially for perpetrators outside the male, heterosexual, and cisgender population. Only one systematic review has focused on the female population of IPV perpetrators, while no systematic or primary studies have identified data regarding the effectiveness of intervention for LGBTQ+ individuals.

Focusing on the geographical aspect, three reviews have systematized evidence from studies conducted in Europe, showing a growing research activity on intervention for IPV perpetration, especially in Spain. Only one systematic review has examined intervention studies conducted in Italy, indicating that research in this context remains limited, and the present umbrella review does not allow for a comprehensive assessment of the state of the art in the Italian territory. Future research could be directed towards studying and evaluating the broader landscape of intervention practices in Italy. Beyond the borders of Italy, the question of the effectiveness of intervention for IPV perpetration remains open. The results produced in 40 years of research highlight the need to turn the page towards interventions with rigorous methodological frameworks, built on the risk and characteristics shown by individuals involved in treatment. The intervention must start from considering the complex, heterogeneous, and contradictory nature of violence in relationships.

### 4.4 Strengths and Limitations of the Umbrella Review

While the results provide important insights, they are limited to studies that met specific inclusion criteria and were available in English or Italian. Consequently, relevant primary studies—especially those published more recently or in other languages—may have been excluded. Furthermore, because the present umbrella review synthesizes evidence at the review level, emerging data from new primary studies are not reflected in this synthesis.

A further limitation concerns the substantial variability in the methodological quality of the included reviews. According to AMSTAR‐2 ratings, many reviews showed low or critically low confidence levels, which constrains the robustness of the conclusions that can be drawn. The evidence base is also characterized by marked heterogeneity in intervention modalities, sample characteristics, and outcome measures—particularly in how recidivism is defined and assessed (e.g., official records vs. partner reports vs. self‐report). This heterogeneity hinders direct comparison across reviews and limits the ability to identify consistent patterns of effectiveness.

Additionally, some degree of overlap in primary studies across reviews is likely, which may inflate the prominence of certain findings within the evidence base. Finally, most primary studies included within the reviews had short follow‐up periods, which restricts conclusions regarding the durability of treatment effects.

Despite these limitations, the umbrella review offers a comprehensive and rigorous overview of existing evidence, synthesizing findings across four decades of research and highlighting both persistent weaknesses and promising directions for future work.

## 5 Conclusions

The synthesis and systematization carried out in this work outline a comprehensive and rigorous overview of the state of the art in the field of intervention for the perpetration of Intimate Partner Violence (IPV). It illustrates both the key findings on intervention effectiveness and the major methodological and contextual limitations that hinder progress in this field.

While this study does not comprehensively answer the questions posed by the author, it shows encouraging results regarding the effectiveness of interventions tailored to the characteristics of their recipients.

While some emerging approaches—such as trauma‐focused, motivational, or substance‐use‐integrated interventions—show promising results, the overall evidence base remains weakened by high attrition, inconsistent outcome measurement, and limited understanding of mechanisms of change. These findings underscore the need for future research to adopt stronger methodological designs, diversify studied populations and cultural contexts, and systematically investigate how, why, and for whom interventions work. By consolidating and critically evaluating the existing review literature, this umbrella review identifies clear priorities for advancing both research and practice in IPV perpetrator intervention.

## Funding

The authors received no specific funding for this work.

## Conflicts of Interest

The authors declare no conflicts of interest.

## Data Availability

The data that support the findings of this study are available from the corresponding author, upon reasonable request.
